# Decreased PRC2 activity supports the survival of basal-like breast cancer cells to cytotoxic treatments

**DOI:** 10.1038/s41419-021-04407-y

**Published:** 2021-11-29

**Authors:** Iga K. Mieczkowska, Garyfallia Pantelaiou-Prokaki, Evangelos Prokakis, Geske E. Schmidt, Lukas C. Müller-Kirschbaum, Marcel Werner, Madhobi Sen, Taras Velychko, Katharina Jannasch, Christian Dullin, Joanna Napp, Klaus Pantel, Harriet Wikman, Maria Wiese, Christof M. Kramm, Frauke Alves, Florian Wegwitz

**Affiliations:** 1 Department of General, Visceral and Pediatric Surgery, University Medical Center Göttingen, Göttingen, Germany; 2 Department of Gynecology and Obstetrics, University Medical Center Göttingen, Göttingen, Germany; 3 Translational Molecular Imaging, Max Planck Institute for Experimental Medicine, Göttingen, Germany; 4 Department of Gastroenterology, GI-Oncology and Endocrinology, University Medical Center Göttingen, Göttingen, Germany; 5 Clinic for Haematology and Medical Oncology, University Medical Center Göttingen, Göttingen, Germany; 6 Institute for Diagnostic and Interventional Radiology, University Medical Center Göttingen, Göttingen, Germany; 7 Institute of Tumor Biology, University Medical Center Hamburg-Eppendorf, Hamburg, Germany; 8 Department of Pediatrics and Adolescent Medicine, Division of Pediatric Hematology and Oncology, University Medical Center Göttingen, Göttingen, Germany

**Keywords:** Breast cancer, Cancer epigenetics, Cancer therapeutic resistance

## Abstract

Breast cancer (BC) is the most common cancer occurring in women but also rarely develops in men. Recent advances in early diagnosis and development of targeted therapies have greatly improved the survival rate of BC patients. However, the basal-like BC subtype (BLBC), largely overlapping with the triple-negative BC subtype (TNBC), lacks such drug targets and conventional cytotoxic chemotherapies often remain the only treatment option. Thus, the development of resistance to cytotoxic therapies has fatal consequences. To assess the involvement of epigenetic mechanisms and their therapeutic potential increasing cytotoxic drug efficiency, we combined high-throughput RNA- and ChIP-sequencing analyses in BLBC cells. Tumor cells surviving chemotherapy upregulated transcriptional programs of epithelial-to-mesenchymal transition (EMT) and stemness. To our surprise, the same cells showed a pronounced reduction of polycomb repressive complex 2 (PRC2) activity via downregulation of its subunits *Ezh2*, *Suz12, Rbbp7* and *Mtf2*. Mechanistically, loss of PRC2 activity leads to the de-repression of a set of genes through an epigenetic switch from repressive H3K27me3 to activating H3K27ac mark at regulatory regions. We identified *Nfatc1* as an upregulated gene upon loss of PRC2 activity and directly implicated in the transcriptional changes happening upon survival to chemotherapy. Blocking NFATc1 activation reduced epithelial-to-mesenchymal transition, aggressiveness, and therapy resistance of BLBC cells. Our data demonstrate a previously unknown function of PRC2 maintaining low *Nfatc1* expression levels and thereby repressing aggressiveness and therapy resistance in BLBC.

## Introduction

Breast cancer (BC) is the most common cancerous disease in women with over 2.2 million new cases in 2020 worldwide, but also represents 1% of all male malignancies [1, 2]. The mortality of BC patients has significantly decreased over the past decades, mostly because of early diagnosis improvements and the development of several targeted therapies. However, despite intensive efforts to combat the disease, BC remains the first cancer-related cause of death among women. Even when early detected, BC recurrence rate fluctuates between 5% and 10% within 10 years [3–6]. Nowadays, around 25% of BC patients still develop resistant and/or metastatic lesions with an unfavorable outcome [7]. Therefore, there is an urgent need for improved treatment options efficiently targeting resistant relapses and metastases.

BC is a remarkably heterogeneous disease. The lesions can be classified into distinct subtypes with specific therapeutic approaches and outcomes, based on the estrogen receptor (ER), the progesterone receptor (PR), and the human epidermal growth factor receptor 2 (HER2) expression [8]. High-throughput gene expression profiling studies led to the definition of at least five different molecular subtypes of BC with very different incidence, prognosis, and response to treatments: Luminal A, Luminal B, HER-2 enriched, BLBC, Normal-like, and Claudin-low [9, 10]. Targeted therapies specifically inhibiting ER, PR, and/or HER2 greatly improved the therapeutic options and prognosis of mammary carcinomas (MaCas) subtypes expressing those receptors. Unfortunately, TNBC patients (~15% of all BC), that largely overlap with the BLBC subtype, lack the expression of hormone and HER2 receptors and do not profit from these therapeutic advances. These lesions are clinically treated with a combination of surgery, radiation, and conventional chemotherapy, depending on the stage of the disease. Despite a good first response to cytotoxic therapies, a large fraction of BLBC patients rapidly develops resistance. Consequently, BLBC shows the highest recurrence rate after treatment and the poorest prognosis among BC diseases [10].

To survive conventional chemotherapeutic treatments, tumor cells need to adapt to new hostile conditions. Acquisition of epithelial–mesenchymal plasticity (EMP) and stemness properties have often been shown to support this process [11, 12]. Such dynamic properties necessitate the presence of epigenetic mechanisms allowing a rapid and reversible reorganization of whole gene expression profiles, rendering them attractive therapeutic targets in cancer [13]. Numerous reports demonstrate the central role of epigenetic factors during epithelial to mesenchymal transition (EMT) [11, 14]. Similarly, epigenetic factors are indispensable for the acquisition and maintenance of cancer stem cell (CSC) properties [14, 15]. This is especially the case for the polycomb repressive complexes 2 (PRC2), a well-characterized epigenetic complex of four core subunits EED, SUZ12, RBBP7, EZH1, or EZH2. PRC2 catalyzes the di- and trimethylation of histone 3 at lysine 27 (H3K27me2 and H3K27me3, respectively) through its catalytic subunit EZH1 or EZH2, promoting chromatin compaction and leading to gene silencing [16, 17]. Interestingly, PRC2 was shown to support adult stem cell homeostasis by repressing differentiation programs, and to promote CSC properties in numerous cancers including BC [18–20]. Furthermore, the enzymatic activity of the PRC2 complex was shown to actively promote EMT by positively regulating the expression of central EMT-transcription factors (EMT-TFs) like SNAI1 or ZEB1 [21, 22].

In the past, we developed and characterized the WAP-T MaCa mouse model to study the biology, progression, and metastatic processes in BLBC [23–28]. WAP-T mice carry the simian virus 40 (SV40) early region under the control of the mammary tissue-specific WAP-promoter, exclusively activated during the lactation. Upon induction through mating, female transgenic animals develop endogenous tumors with strong CSC properties and phenotypic plasticity [26, 29, 30]. In a former effort to understand the effects of conventional cytotoxic combination therapy (cyclophosphamide, anthracycline, and 5-fluorouracil; short CAF) on BC, we observed that the chemotherapeutic treatment was not able to eradicate the disease in vivo, recapitulating the clinical situation. Interestingly, surviving tumor cells displayed a more aggressive mesenchymal-like phenotype with increased stem cell traits and showed a pronounced tendency to disseminate [31]. In the present study, we established an in vitro approach to interrogate the molecular mechanisms underlying the acquisition of EMP and stemness allowing murine WAP-T and human BLBC cells to survive the chemotherapy. BC patient material and in vivo experiments were finally used to support and validate our findings. Collectively, we identified a previously unknown PRC2-mediated repressive function exerted on EMT- and CSC programs by suppressing the expression of the nuclear factor of activated T cells 1 (*NFATC1*) in BLBC cells.

## Results

### WAP-T cells surviving a conventional cytotoxic combination therapy (CAF) gain stem cell traits and EMT properties in vitro

**Fig. 1:**
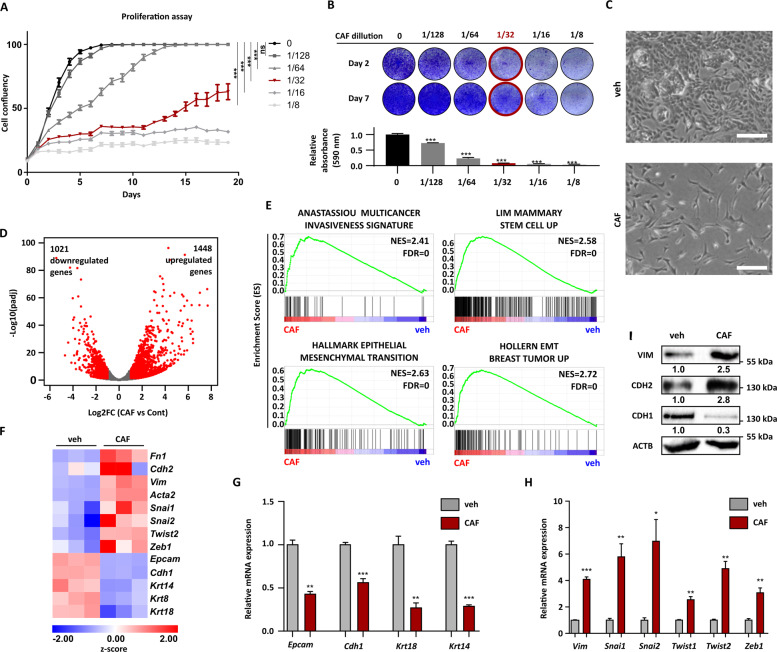
WAP-T cells surviving a conventional cytotoxic combination therapy (CAF) gain stem cell traits and EMT properties in vitro. **A** Cell proliferation assay of pG-2 cells treated for 48 h with increasing concentrations of a combinatory CAF chemotherapy. The concentrations represent a fraction of the dose used in our previous publication [31]. Cell confluency was assessed every day with a Celigo^®^. **B** Crystal violet staining of pG-2 cells performed on days 2 and 7 after different doses of CAF-chemotherapy. A quantification (absorbance at 590 nm) of solubilized crystal violet at day 7 is provided in the lower panel. **C** Phase contrast images of pG-2 cells 48 h after CAF-treatment showing a spindle-like morphology characteristic for cells that underwent EMT (scale bar = 250 µm). **D** Volcano plot showing transcriptome-wide gene expression changes in pG-2 cells treated with CAF for 48 h compared to vehicle. The number of regulated genes regulated upon CAF-treatment in the mRNA-seq analyses treatment was assessed using the following parameters: |log2FC | > 1 and p-adj < 0.05. **E** Representative GSEA enrichment plots showing significant enrichment of gene signatures characteristic for EMT-processes, stemness traits, and cancer aggressiveness in CAF-treated versus control pG-2 cells. **F** Heatmap showing the regulation of selected EMT markers identified in the mRNA-seq analyses in CAF-treated versus control pG-2 cells. **G** and **H** qRT-PCR results validating the downregulation of epithelial marker (**G**) and the upregulation of mesenchymal marker (**H**) upon CAF-treatment of pG-2 cells. Data were calibrated on the control condition and normalized on the *Rplp0* housekeeping gene. **I**: Western blot validating an increase of Vimentin and N-cadherin protein levels as well as a decrease of E-Cadherin protein levels upon 48 h CAF treatment of pG-2 cells. Error bars: standard error of the mean (SEM), *p-val ≤ 0.05, **p-val ≤ 0.01, ***p-val ≤ 0.005, One-way ANOVA: **A** (AUC: area under the curve), **B** Student’s *t*-test: **G**, **H** All experiments were performed in at least biological triplicates (*n* = 3).

To identify the molecular mechanisms underlying the survival and the emergence of resistance to CAF chemotherapy in vitro, we first optimized the treatment settings of a well-characterized WAP-T cell line (G-2 cells) in the cell culture [29]. The aim here was the identification of treatment conditions eradicating most of the tumor cells but allowing the survival and regrowth of a small tumor cell fraction, mimicking, thereby, the in vivo relapse situation. Combination therapy consisting of 312.5 ng/ml cyclophosphamide, 15.6 ng/ml doxorubicin, and 312.5 ng/ml 5-FU, corresponding to the 1/32 dilution of the therapy previously utilized in Jannasch et al. [31], was identified as the most appropriate setting (Fig. 1A and B). Therefore, this treatment was adopted for the rest of the in vitro experiments of the present study (designated as CAF). Interestingly, parental G-2 (pG-2) cells surviving CAF treatment acquired a more elongated morphology characteristic for EMT-undergoing cells (Fig. 1C). A chemoresistant variant of the pG-2 cells called resistant G-2 (rG-2) cells was established through several cycles of CAF treatments (see the “Methods” section). rG-2 harbor cells at basal state a mesenchymal-like phenotype, further supporting the implication of EMT-mechanisms in CAF-resistance (Fig. S1A and S1B). We compared the transcriptome of pG-2 cells after 48 h of CAF treatment to vehicle conditions (veh) using mRNA-sequencing (mRNA-seq). DESeq2 analyses identified 1021 downregulated and 1448 upregulated genes (|Log2(Fold Change)| > 1, *p*-adj < 0.05) in CAF-treated cells (Fig. 1D). Gene set enrichment analyses (GSEA) revealed strong enrichment of EMT-gene sets, cancer aggressiveness, and stemness (Fig. 1E). Indeed, well-known EMT markers (*Vim*, *Cdh2, Fn1*, and *Acta2*) and EMT-TFs (*Snai1*, *Snai2*, *Twist2*, and *Zeb1*) were upregulated in surviving cells whereas the expression of epithelial markers (*Cdh1*, *Epcam, Krt14, Krt8*, and *Krt18*) was strongly reduced (Fig. 1F). The regulation of selected epithelial (*Epcam, Cdh1, Krt18*, and *Krt14*) and mesenchymal genes (*Vim*, *Snai1*, *Snai2*, Twist1, *Twist2*, *Zeb1*) was validated via qPCR (Fig. 1G and H). We confirmed the increased protein levels of Vimentin and N-cadherin as well as the decrease of E-cadherin via western blot (Fig. 1I). Interestingly, the expression of stem cell-specific transcription factors (e.g. *Sox2* and *Nanog*) was also found to be increased in CAF-treated pG-2 cells (Fig. S1C). Similarly, at basal state, rG-2 cells showed increased expression of several EMT- and stem cell-markers compared to pG-2 cells (Fig. S1C). Altogether, these results strengthened the validity of our in vitro approach mimicking our previous in vivo studies [31] and further emphasize the implication of EMT- and stem cell properties in chemotherapy survival mechanisms.

### WAP-T tumor cells surviving CAF treatment downregulate the expression of PRC2 core subunits

**Fig. 2:**
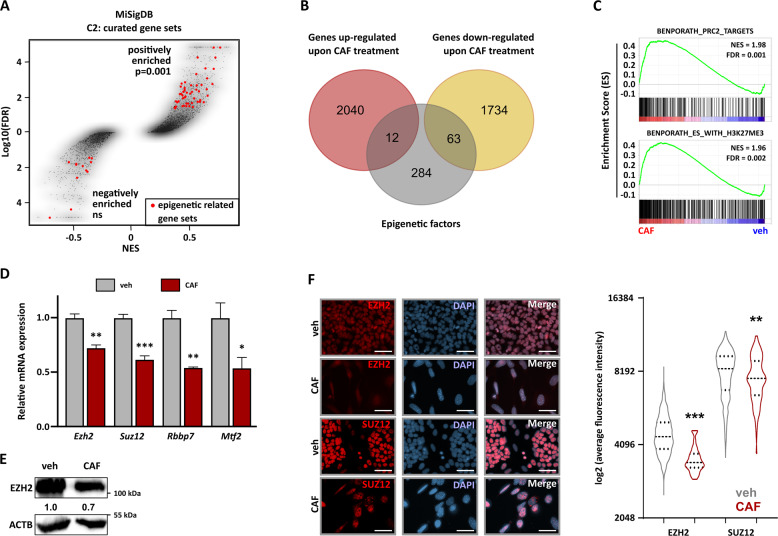
WAP-T tumor cells surviving CAF treatment downregulate the expression of PRC2 core subunits. **A** GSEA analysis results (MiSigDB C2: curated gene sets) plotted as an overview along with Normalized Enrichment Score (NES) and log10(FDR). The red dots represent significantly enriched epigenetic-related gene sets. A significant positive enrichment of gene signatures associated with epigenetic mechanisms perturbation was identified. **B** Identification of differentially regulated epigenetic factors: genes regulated in pG-2 cells upon survival to CAF treatment (|Log2(Fold Change)| > 0.7, *p*-adj < 0.05) were intersected with a list of known epigenetic factors. No significant enrichment of epigenetic factors was observed in the groups of up or down-regulated genes, as assessed by Fisher’s exact test. **C** Representative GSEA enrichment plots showing the enrichment of gene signatures typically repressed by PRC2 in CAF-treated pG-2 cells. **D** Validation of *Ezh2*, *Suz12*, *Rbbp7*, and *Mtf2* expression changes via qRT-PCR. Data were calibrated on the control condition and normalized to the *Rplp0* housekeeping gene expression. **E** Reduction of EZH2 protein levels upon CAF treatment assessed via western blot. **F** Immunofluorescence staining showing a reduction of EZH2 and SUZ12 levels upon CAF treatment of pG-2 cells (scale bars = 50 µm). A quantification of EZH2 and SUZ12 signals is provided in the right panel. Error bars: standard error of the mean (SEM). *p-val ≤ 0.05, **p-val ≤ 0.01, ***p-val ≤ 0.005. Statistical tests: Fisher exact test (**A**), Student’s *t*-test (**D**), Mann-Whitney test (**F:** violin plot). All experiments were performed in biological triplicates (*n* = 3).

Further GSEA analyses identified an accumulation of epigenetic-regulatory gene signatures in CAF-treated cells (Fig. 2A). This was an interesting finding, as several epigenetic mechanisms are involved in processes controlling cellular plasticity [32]. mRNA-seq identified 63 down-regulated and 12 up-regulated epigenetic factors (Fig. 2B, listed in Table S1). Notably, GSEA and Enrichr analyses pointed at the regulation of genes known to be H3K27me3-marked and/or repressed by PRC2 (Figs. 2C and S2A). A closer look at core PRC2 subunits expression revealed the downregulation of *Ezh2*, *Suz12*, *Rbbp7*, and the classical accessory factor *Mtf2* in pG-2 cells surviving CAF treatment (Fig. 2D). Also on the protein level, EZH2 and SUZ12 were significantly reduced in the CAF condition, as assessed via western blots and immunofluorescence staining (Figs. 2E, F, S2C, D). In line with these findings, rG-2 cells grown under normal conditions harbored a constant lower expression of the core PRC2 subunits *Ezh2*, *Suz12*, *Rbbp7*, and *Mtf2* compared to untreated or treated pG-2 cells. Noticeably, their expression levels were even more reduced upon CAF treatment (Fig. S2B). We concluded that the reduction of PRC2 levels was associated with the survival to cytotoxic therapies and chemotherapy-resistant phenotypes.

### Reduction of EZH2 activity enhances the aggressiveness of BLBC tumor cells

**Fig. 3:**
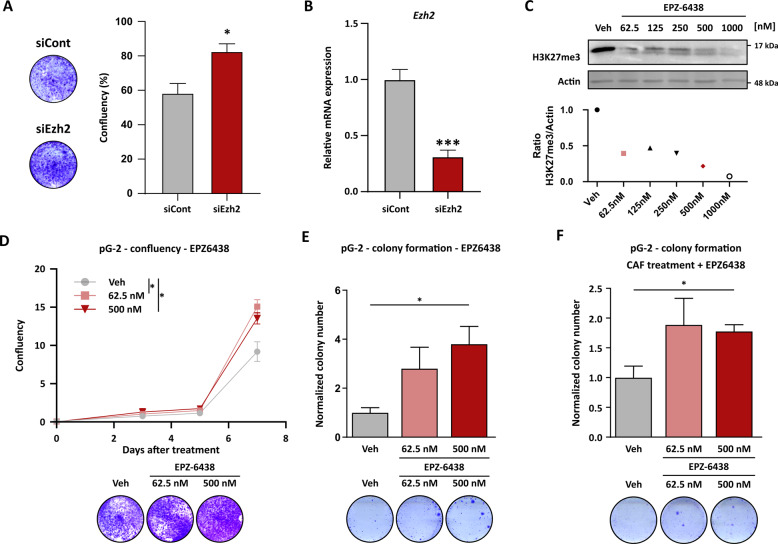
Reduction of EZH2 activity enhances the aggressiveness of BLBC tumor cells. **A** Crystal violet staining of pG-2 cells with EZH2 knockdown. The confluency was measured by ImageJ and normalized to the controls. **B** Validation of EZH2 knockdown efficiency via qRT-PCR. Gene expression was calibrated to the control condition (siCont) and normalized to the *Rplp0* housekeeping gene. **C** Measurement of EZH2 inhibition by increasing EPZ-6438 concentration via assessment of H3K27me3 levels in western blots. Densitometry normalized to actin levels is provided in the lower panel. **D** Proliferation assay of EPZ-6438-treated pG-2 cells using celigo^®^ and crystal violet staining. **E** and **F** Colony formation assay upon treatment of pG-2 cells with EPZ-6438 alone (**E**) or in combination with CAF (**F**). The number of colonies was assessed through ImageJ analysis. Error bars: standard error of the mean (SEM). *p-val ≤ 0.05, **p-val ≤ 0.01, ***p-val ≤ 0.005. Student’s *t*-test: **A** and **B** one-way ANOVA: **D–F** All experiments were performed in biological triplicates (*n* = 3).

Although PRC2 was so far majorly associated with tumor-promoting functions, recent publications have also pointed toward a possible tumor-suppressive role, for example in ovarian carcinoma [33]. Therefore, we asked whether the identified reduction of PRC2 activity could directly mediate WAP-T tumor cell survival to cytotoxic therapies by de-repressing specific gene expression programs involved in tumor aggressiveness and/or proliferation. To assess the effect of EZH2 activity loss on the proliferation of pG-2 cells, we silenced *Ezh2* using targeted siRNA or treatment with a small molecule inhibitor against EZH2 (EPZ-6438). Interestingly, impairment of EZH2 activity did not reduce the proliferation of the tumor cells as it was observed for numerous other BC cell lines in the past [34, 35]. On the contrary, the growth of pG-2 cells was slightly but significantly increased by the loss of EZH2 activity upon knockdown (Fig. 3A) and at a low (500 nM) and very low (62.5 nM) concentration of EPZ-6438 (Fig. 3D). EZH2 knockdown efficiency was validated on mRNA level (Fig. 3B) and gradual loss of H3K27me3 resulting from EPZ-6438 treatment was measured by western blot for different concentrations (Fig. 3C). The treatments of pG-2 cells with 62.5 and 500 nM EPZ6438 induced a reduction of 60% and 80% of the H3K27me3 signal, respectively. Interestingly, the colony formation capacity of pG-2 cells seeded at low cell density was strongly improved upon EZH2 inhibition, suggesting increased tumor-initiating properties (Fig. 3E). Remarkably, this increased colony formation capacity was maintained upon chemotherapy treatment, indicating that inhibition of PRC2 complex activity indeed supported cell survival and resistance to the therapy (Fig. 3F). We asked whether this observation was limited to the murine WAP-T MaCas or if other human cancer cell lines could also get growth and survival advantage upon PRC2 activity loss. Although certain BC cell lines showed impaired or unchanged proliferation upon EZH2 inhibition, knockdown of EZH2 in the MDA-MB-468 TNBC cell line stimulated the growth properties of the cells, with an even more pronounced effect under CAF treatment (Fig. S3A and B). Noticeably, the increased proliferation upon EZH2 inhibition was also observed in human cancer cell lines of other origins, such as colorectal and bile duct carcinoma (Fig. S3C–F). Together, our data showed that PRC2 inhibition can increase aggressiveness and cytotoxic therapy survival of cancer cells in a context-dependent manner.

### Reduction of PRC2 activity during chemotherapy treatment allows activation of gene expression programs promoting tumor cell survival

**Fig. 4:**
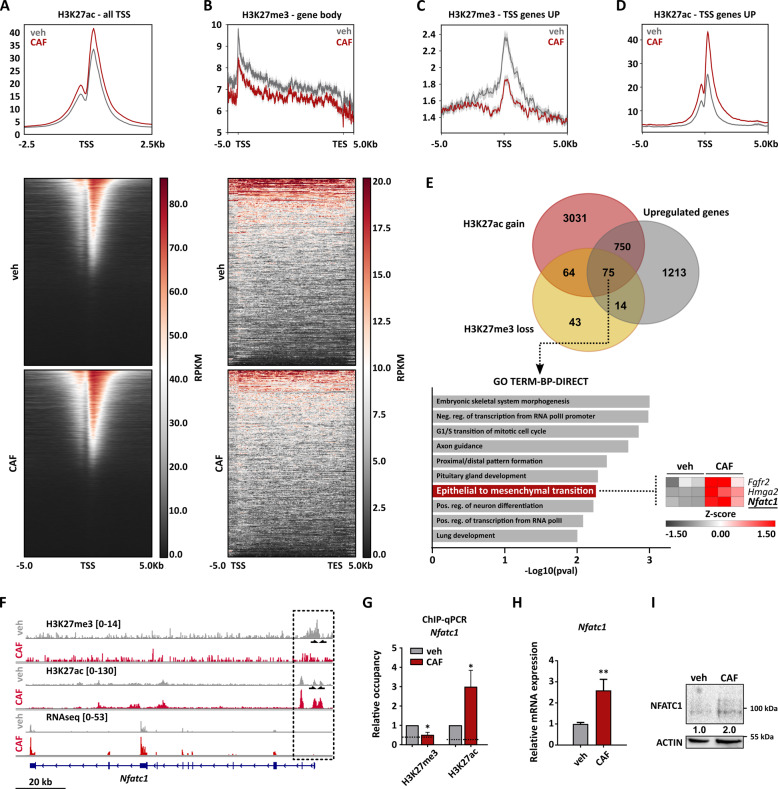
Reduction of PRC2 activity during chemotherapy treatment allows activation of gene expression programs promoting tumor cell survival. **A** Global changes of H3K27ac at TSSs (±2.5 kb) upon CAF-treatment, visualized in an aggregate plot and heatmaps. **B** Global changes of H3K27me3 in gene bodies (±5 kb) of genes significantly marked under basal growth conditions, visualized in an aggregate plot and heatmaps. **C** and **D** Aggregate plots showing changes of H3K27me3 (**C**) and H3K27ac (**D**) occupancy at TSSs (±5 kb) of genes upregulated (log2FC > 0.8, *p*-val < 0.05) upon 48 h CAF-treatment. **E**: Identification of up-regulated genes showing a simultaneous loss of H3K27me3 levels and gain of H3K27ac occupancy upon CAF-treatment (upper panel). This group of genes was statistically significantly enriched, as assessed by Chi-square test. The 75 gene set was then used for signature enrichment analyses. Interestingly, regulators of the EMT program were identified in the Gene Ontology (GO) databases (DAVID web tool, (link: https://david.ncifcrf.gov/). **F** H3K27me3 and H3K27ac ChIP-seq tracks, and mRNA-seq tracks showing specific occupancy changes upon CAF treatment at the *Nfatc1* gene locus. The position of the primers used in the subsequent ChIP-qPCRs is provided by black arrows. **G** Validation of the epigenetic switch at the promoter region of *Nfatc1* via ChIP-qPCR. The dashed line represents the background signal obtained with IgG control. **H** and **I** Changes of *Nfatc1* expression upon CAF-treatment, as measured via qRT-PCR (**H**) and western blot (**I**). The densitometry values represent the normalized NFATc1/Actin signal. Error bars: Standard error of the mean (SEM). *p-val ≤ 0.05, **p-val ≤ 0.01, ***p-val ≤ 0.005. Student’s *t*-test: **G**, **H** All experiments were performed in at least biological triplicates (*n* = 3).

Loss of PRC2 activity during chemotherapy survival could lead to an epigenetic switch enabling tumor cells to activate expression programs promoting aggressiveness and therapy resistance. To test this hypothesis, we assessed genome-wide occupancy of H3K27me3 and H3K27ac via ChIP-seq in treated and untreated pG-2 cells. The analysis of genome-wide H3K27ac peak changes showed that cytotoxic treatment of pG-2 cells leads to a genome-wide signal increase, especially at transcription start sites (TSSs), whereas only a minority of regions showed a reduction (Figs. 4A and S4A). This result aligns with the observation that chemotherapy broadly leads to gene up-regulation, as shown in our previous mRNA-seq analysis (Fig. 1D), and further supports the potential occurrence of global transcriptional de-repression caused by a PRC2 activity reduction. Therefore, we analyzed changes of H3K27me3 occupancy upon CAF treatment. Interestingly, we observed a mild but significant genome-wide decrease in H3K27me3 signal (Fig. S4B). Notably, the observed H3K27me3 signal decrease upon chemotherapy treatment was found even more pronounced in actively repressed gene bodies by the PRC2 under basal conditions (Fig. 4B). We focused on changes of epigenetic state at the TSSs of upregulated genes. Interestingly, average levels of H3K27me3 at TSS of up-regulated genes were pronouncedly reduced upon treatment (Fig. 4C). In parallel and as expected, H3K27ac occupancy strongly increased at TSSs of up-regulated genes (Fig. 4D). We, therefore, suspected a direct connection between loss of PRC2 repressive activity and activation of gene expression programs upon CAF treatment. To confirm our assumption, we decided to identify the subset of up-regulated genes directly controlled through a H3K27me3-to-H3K27ac epigenetic switch (Fig. 4E). This analysis identified 139 genes with a switch from trimethylation to acetylation at H3K27. Strikingly, 75 genes showed at the same time a robust up-regulation at the RNA level (Log2FC > 0.7, *p*-val < 0.05). As EMT was identified as one of the major features of cells surviving CAF-treatment, we screened for EMT-master regulators in gene ontology and pathway analyses using the DAVID database. We identified here the nuclear factor of activated T-cells cytoplasmic 1 (*Nfatc1*), the High Mobility Group AT-Hook 2 (*Hmga2*), and fibroblast growth factor receptor 2 (*Fgfr2*) as being significantly enriched (Fig. 4E). Changes in epigenetic profiles were visualized for these three genes (Figs. 4F and S4C) and validated by ChIP-qPCR (Figs. 4G and S4D). Changes in *Nfatc1*, *Fgfr2* and *Hmga2* expression levels were further validated by qRT-PCR and western blot (Figs. 4H–I, S4E and S4F). *Nfatc1* retained our attention as it was shown to promote EMT and tumor progression in several tumor entities. Furthermore, Chen et al. reported a context-dependent epigenetic regulation of NFATc1 expression by EZH2 in pancreatic tissues [36]. Additionally, NFATc1 activity can be targeted by small molecule inhibitors, some of them being commonly employed in the clinic (e.g. Cyclosporin A, CsA), making this factor very attractive to study in the context of survival and resistance to chemotherapy [37].

### EZH2 loss stimulates *NFATc1* expression and correlates with poor prognosis in BLBC patients

**Fig. 5:**
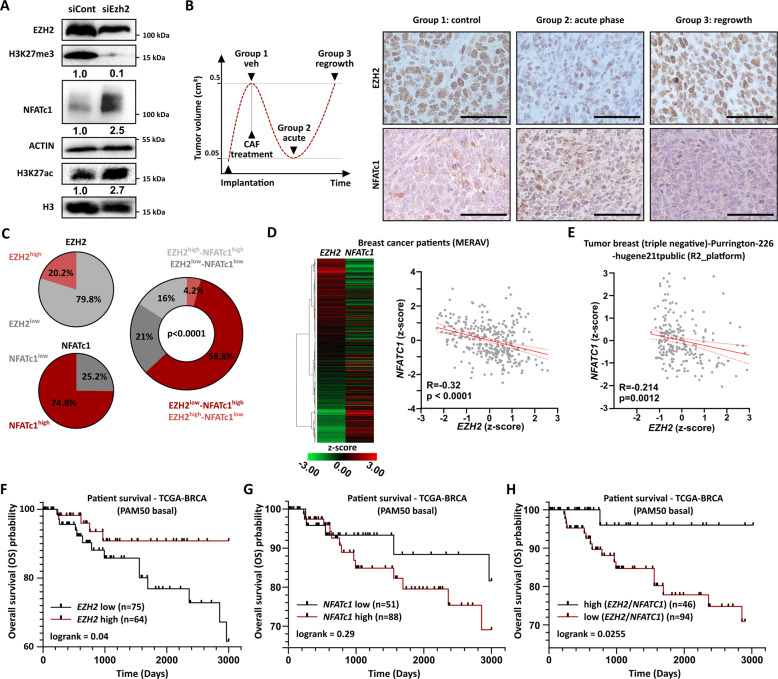
EZH2 loss stimulates *NFATc1* expression and correlates with poor prognosis in BLBC patients. **A** siRNA-mediated EZH2 knockdown leads to a global loss of H3K27me3 and increased NFATc1 protein levels in pG-2 cells, as assessed by western blot. The densitometry values provided here represent H3K27me3/H3, NFATc1/Actin, and H3K27ac/H3 signals, respectively. **B** Schematic representation of the groups of CAF-treated tumors from Jannasch et al. [31] (left panel). Representative images of paraffin-embedded tumors from group 1 (control), group 2 (acute phase), and group 3 (regrowth phase) stained for EZH2 and NFATc1 (right panel). Scale bar = 50 µm. **C** Expression levels of EZH2 and NFATc1 alone (left charts) and anti-correlated with each other (right chart) in BC TMA samples (*n* = 119). **D** and **E** Distribution of *EZH2* and *NFATc1* expression levels in Metabolic gEne RApid Visualizer database (**D**; MERAV; http://merav.wi.mit.edu) and R2: Genomics Analysis and Visualization Platform (**E**; https://hgserver1.amc.nl/cgi-bin/r2/main.cgi). A significant anti-correlation between *EZH2* and *NFATc1* expression was observed (**D** and **E**). **F–H** Survival analyses of BLBC patients (PAM50 classification) showing overall survival (OS) advantage for the *EZH2*^high^ (**F**) or *EZH2*^high^/*NFATC1*^low^ (**H**) and a poorer survival for the *NFATC1*^high^ (**G**) and *EZH2*^low^/*NFATC1*^high^ (**H**) -expressing groups (**H:** based on the *z*-score (EZH2)/z-score(NFATc1) ratio). Expression and survival data of BLBC patient data were extracted from the TCGA-BRCA dataset on the Xena browser (link: https://xenabrowser.net/). Statistical tests: Fisher exact test (**C**), Pearson correlation (**D**, **E**), Log-rank test (**F–H**). All experiments were performed in at least biological triplicates (*n* = 3).

Next, we investigated whether EZH2 directly modulates NFATc1 expression in WAP-T cells. We, therefore, performed a knockdown of EZH2 in pG-2 cells followed by western blot analyses. As expected, EZH2 loss induced a global decrease of H3K27me3 levels. Interestingly, the global levels of H3K27ac were increased, confirming the occurrence of a profound epigenetic switch upon PRC2 activity loss, as observed earlier in our ChIP-seq results (Figs. 4A, B, S4A, and B). Strikingly, loss of EZH2 induced a dramatic increase of NFATc1 at the protein level, demonstrating that the sole impairment of PRC2 activity suffices to promote *Nfatc1* expression in vitro (Fig. 5A). Accordingly, EPZ-6438 mediated EZH2 inhibition in pG-2, rG-2 and MDA-MB-468 cells also led to increased NFATc1 levels, while EZH2 overexpression in pG-2 cells led to a significant reduction of *Nfatc1* expression, demonstrating the validity of the EZH2-mediated repressive activity on NFATc1 expression in other models (Fig. S5A–E). We next analyzed the behavior of EZH2 and NFATc1 in vivo on paraffin sections of WAP-T tumors at different time points of a CAF chemotherapy treatment generated in our former study [31] (Fig. 5B). IHC staining confirmed a strong decrease of EZH2 levels in surviving tumor cells during the acute phase of the treatment (Group 2) compared to the untreated control group (Group 1). Simultaneously, the number of cells expressing NFATc1 as well as its staining intensity were increased in Group 2 tumors, confirming the anti-correlative behavior of these two factors in vivo. Interestingly, tumors re-growing after chemotherapy (Group 3) showed an almost complete restoration of EZH2 expression whereas NFATc1 expression came back to a level close to the control group (Group 1) (Figs. 5B and S5F). Additionally, we asked if the transcriptional control of *NFATc1* by EZH2 could be observed in BC patient material. The staining of a BC tumor microarray (TMA) with 119 different samples revealed that the majority of the tumor samples expressed moderate levels of EZH2 and high levels of NFATc1 (Fig. 5C left panel and Fig. S5G). Strikingly, an anti-correlation between EZH2 and NFATc1 levels was observed in ~2/3 of the samples (Fig. 5C, right panel). To further support our finding, we extracted the expression values of *EZH2* and *NFATc1* from different publicly available human primary BC datasets and observed a mild but significant anti-correlation (Fig. 5D, E). Survival analyses using the TCGA PAM50-based dataset for human basal-like cancers showed that patients with low *EZH2* (Fig. 5F), high *NFATC1* (Fig. 5G) or low (*EZH2*/*NFATC1*)-ratio (Fig. 5H) have a worse prognosis compared to *EZH2* high, *NFATC1* low or high (*EZH2*/*NFATC1*)-ratio patients. Additional mining of publicly available data revealed that TNBC patients with low response to chemotherapy show significantly lower levels of *EZH2* and higher *NFATc1* expression (Fig. S5H and I). Therefore, *NFATC1* induction by *EZH2* loss is implicated in increased tumor aggressiveness and progression in BLBC patients.

### Inhibition of NFATc1 sensitizes BLBC to conventional chemotherapy

**Fig. 6:**
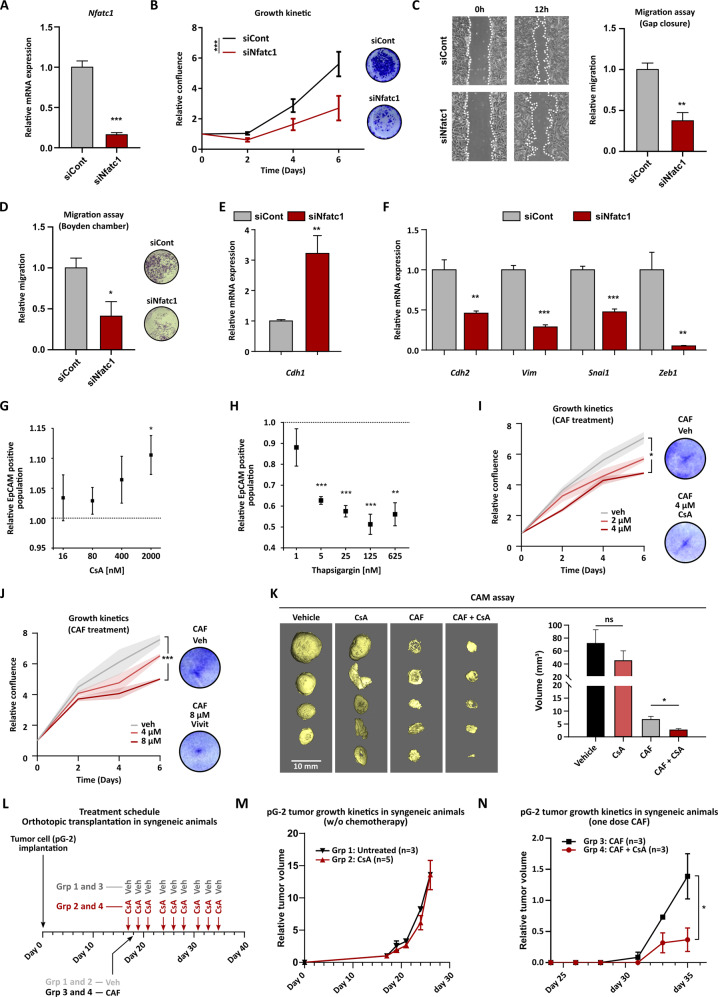
Inhibition of NFATc1 sensitizes BLBC to conventional chemotherapy. **A** qRT-PCR validation of NFATc1 knockdown. **B** Impairment of pG-2 cell growth upon NFATc1 knockdown determined by Celigo^®^ (left panel) and crystal violet staining (right panel). **C** and **D** Reduction of pG-2 cells migratory capacity upon NFATc1 knockdown analyzed via gap closure assay (**C**) and Boyden chamber assay (**D**). **E** and **F** Assessment of EMT markers regulation in pG-2 cells upon NFATc1 knockdown by qRT-PCR. **G** The inhibition of NFATc1 activity by treating pG-2 cells with increasing concentrations of cyclosporin A (16, 80, 400 nM, and 2 µM) for 48 h increases the fraction of EpCAM positive cells, as measured by FACS. **H** Activation of the NFAT signaling through thapsigargin treatment (1, 5, 25, 125, and 625 nM) for 48 h reduces the fraction EpCAM-positive cells in pG-2 cells, as measured by FACS. **I** and **J** Inhibition of NFATc1 transcriptional activity by Cyclosporin A (CsA) (**I**) or VIVIT (**J**) treatment renders pG-2 cells more sensitive to CAF chemotherapy in vitro. Growth kinetics were assessed by Celigo^®^ confluence measurement (left panel) and crystal violet staining (right panel). Error bars depicted as shadow area: standard error of the mean (SEM). **K** CAM assay demonstrating a significantly decreased size of the tumors treated with a combination of CAF + CsA. The Micro-CT scans of the tumors and quantification of their volumes are provided for the different conditions in the left and right panels, respectively. **L–N** Assessment of the CAF + CsA combination therapy efficiency in in vivo syngeneic mice transplanted with pG-2 cells. Graph depicting the treatment schedule for the different groups of mice (**L**). Growth kinetics of untreated (*n* = 3 animals) or CsA (50 mg/kg i.p. three times per week, *n* = 5 animals) treated tumors (**M**). Growth kinetics of tumors treated with either CAF alone (*n* = 3 animals) or CAF + CsA in combination (*n* = 3 animals) (**N**). *p < 0.05, **p < 0.001, ***p < 0.005. Student’s *t*-test: **A**, **B**, **N** (AUC: area under the curve), **C**–**F**, **I**, **J**, **N**. One-way ANOVA: **G**, **H**, **K**. All experiments were performed in at least biological triplicates (*n* = 3).

The NFAT transcription factor family has been shown to regulate EMT processes in different cancers, including BC [38, 39]. Therefore, we decided to investigate the impact of NFATc1 on pG-2 cell aggressiveness. For this purpose, we performed several in vitro functional assays upon siNfatc1 treatment. The knockdown efficiency was first confirmed via western blot and qRT-PCR (Figs. 6A and S6A). Cell growth kinetic measurements demonstrated that NFATc1 loss decreases pG-2 cell growth properties (Fig. 6B). Remarkably, NFATc1 silencing in the human MDA-MB-468 induced an even more pronounced impairment of cell proliferation (Fig. S6B). The treatment of pG-2 cells with CAF leads to increased tumor cell dissemination and EMT in vivo [31]. To determine if increased NFATc1 levels could be involved in these phenomena, we assessed changes of pG-2 cell motility upon loss of NFATc1. We observed that pG-2 cell migratory properties were significantly reduced upon NFATc1 knockdown in a gap closure and a trans-well assay (Fig. 6C and D). Thus, we asked if NFATc1 could influence pG-2 motility by regulating EMT-transcriptional programs. Thus, we performed qRT-PCRs for several EMT-related markers that were previously found to be regulated upon chemotherapy treatment (Fig. 1G and H). Strikingly, NFATc1 loss led to a significant reduction of the EMT signature, as visible by increased levels of epithelial *Cdh1* and decreased EMT-factors *Chd2*, *Vim*, *Snai1*, and *Zeb1* expression (Fig. 6E and F). To confirm that NFATc1 signaling impairment indeed favors more epithelial phenotypes, we treated pG-2 cells with increasing concentration of cyclosporine A (CsA), a well-established inhibitor of NFAT family members. We then measured changes of the epithelial population size by assessing EpCAM-positive (EpCAM^pos^) cell fraction by flow cytometry. Interestingly, CsA treatment of pG-2 cells led to a pronounced increase of the EpCAM^pos^ cell population (Fig. 6G). Remarkably, activation of the NFAT signaling by Thapsigargin treatment induced a strong concentration-dependent decrease of the EpCAM^pos^ cell population, pointing at an efficient induction of EMT upon NFATc1 activation. (Fig. 6H). To finally test if the modulation of NFATc1 activity has an influence on cell’s resistance to chemotherapy, we treated pG-2 with increasing concentrations of CsA and another NFAT-specific inhibitor (VIVIT), in presence or absence of CAF treatment. Both treatments significantly reduced the growth capacity of G-2 cells under basal conditions (Fig. S6C and D). Remarkably, the combination of NFATc1 inhibition and chemotherapy significantly increased the efficiency of the treatment, demonstrating that NFATc1 plays a critical role during survival of tumor cells to cytotoxic treatments (Fig. 6I and J). These effects were not limited to the murine cell line, as CsA treatment also sensitized MDA-MB-468 cells to CAF therapy (Fig. S6E). Finally, we assessed the ability of CsA to inhibit pG-2 cell growth with or without CAF in chorio-allantoic membrane (CAM) assay and in in vivo syngeneic mice (Fig. 6K–N). Interestingly, the single CsA treatment was not able to reduce the growth behavior of the tumors in both assays. However, the combined CsA and CAF therapy showed the highest effectiveness in the CAM assay and in orthotopic growing lesions (Fig. 6K and N). Together, our results demonstrate a key-role of NFATc1 signaling modulating EMP, aggressiveness and chemotherapy survival in pG-2 cells, under the control of PRC2 epigenetic regulation.

## Discussion

In the present study, we leveraged murine WAP-T MaCa cells and human BC-cell lines to model and investigate molecular mechanisms underlying BLBC survival to conventional chemotherapy. Similarly to former studies on in vitro, animal models, and patient material [40, 41], our transcriptome-wide analyses showed that WAP-T cells activate transcriptional programs characteristic for EMT and CSC to survive the treatment. Indeed, the gain of EMP was shown to promote tumor cell invasiveness and protect them against pro-apoptotic signals [42, 43]. Additionally, EMT and CSC properties are tightly linked together and have been frequently shown to positively influence their respective transcriptional programs [44–47]. As the acquisition of such properties requires rapid and profound transcriptional changes, we expected epigenetic mechanisms to be involved in these processes [48]. Combining mRNA-seq and ChIP-seq approaches, we surprisingly identified a reduction of the PRC2/EZH2 activity occurring during chemotherapy survival in WAP-T cells. The repressive activity of EZH2 on gene expression is mostly known to promote cancer progression and contribute to therapy resistance, to metastasis and resistance to programmed cell death in numerous cancers including TNBC [49–60]. Paradoxically, our results unraveled an opposite role of PRC2/EZH2 in WAP-T and other BLBC cells, maintaining a more chemotherapy-sensitive phenotype via specific repression of EMT and CSC transcriptional programs. Although contradictory at the first glance, our results align with still scarce but growing evidence, that loss of PRC2/EZH2 activity can drive or support the initiation and progression of cancers in a context-specific manner [61–63]. For instance, loss of EZH2 was shown to promote genomic instability, to act as a barrier to KRAS-driven inflammation and EMT or to promote therapy resistance in BC, colorectal cancer, lung cancer, acute myeloid and T-cell acute lymphoblastic leukemia, respectively [62, 64–70]. Our investigations on murine and human BLBC cell lines corroborated the occurrence of EZH2-specific tumor-suppressive activity and described thereby a new molecular mechanism by which PRC2/EZH2 can exert its repressive function on the EMT transcriptional program. Specifically, loss of PRC2 subunits upon chemotherapeutic treatment led to rapid upregulation of central EMT regulators via a repressive (H3K27me3) to activating (H3K27ac) epigenetic switch. Strikingly, we identified here NFATc1 as one of the major EMT-TF under the immediate epigenetic control of PRC2 in BLBC and upregulated in cells surviving chemotherapy. Interestingly, the Hessmann group reported a few years ago that NFATc1 is needed for the pancreas regeneration after injury, and is epigenetically silenced by EZH2 activity once regeneration is completed, supporting the mechanism of regulation identified in the present study [36]. The pivotal role of NFATc1 in the activation of EMT transcriptional programs in cancer cells and the availability of specific small molecule inhibitors (e.g. CsA or VIVIT) renders this factor very interesting as a potential drug target to increase conventional therapies efficiency [71, 72].

NFAT-signaling was established as being crucial for the survival and metastatic properties of triple-negative BC [73]. In this study, we observed increased efficiency of CAF treatment on BLBC cells when co-treated with cyclosporine A or VIVIT. These results are in line with former studies on lung cancer, acute myeloid leukemia (AML), and bladder cancer showing that NFATc1 inhibition sensitized cancer cells to cisplatin, sorafenib- and tacrolimus-induced apoptosis, respectively [74–76]. Beside these considerations, NFATc1 has a prominent function in T-cells induction and is necessitated for the correct activation of cytotoxic T cells during tumor cell clearance [77]. The reversible character of NFATc1 inhibitors like CsA as well as their anti-tumorigenic properties at significantly lower doses than immunosuppressive ones, might therefore represent a major advantage in the design of combinatory therapies [37].

In summary, this study presents the evidence of a very context-dependent PRC2/EZH2 function in BC that in certain circumstances can maintain more therapy-sensitive states by epigenetically repressing NFATc1 expression. Our data suggest that targeting the NFATc1 signaling in EZH2^low^ TNBC/BLBC patients could represent an attractive opportunity to increase the efficiency of conventional chemotherapeutic treatments and reduce the development of deadly resistant cancer cells phenotypes.

## Materials and methods

### Cell culture

#### Cell lines

The murine MaCa pG-2 cell line was generated in a previous study^29^ and cultured in DMEM GlutaMAX™ (Invitrogen). The human cell line MDA-MB-468 (triple-negative BC) was cultured in DMEM GlutaMAX™ medium, HCT116 and HT29 (colorectal cancer) in McCoy’s medium (Gibco), and EGI-1 and TFK1 (cholangiocarcinoma) in MEM medium (Gibco). All media were supplemented with 10% fetal bovine serum (FBS) and 1% penicillin/streptomycin (P/S) and all cell lines were maintained at 37 °C, 5% CO_2_.

#### Chemotherapy treatment

Optimization steps were started with a CAF-chemotherapy concentration of 10 μg/ml cyclophosphamide, 0.5 μg/ml doxorubicin, and 10 μg/ml 5-FU, related as concentration “1” [31]. A dilution of “1/32” (312.5 ng/ml cyclophosphamide, 15.6 ng/ml doxorubicin, and 312.5 ng/ml 5-FU) was identified as most appropriate for this study and was employed in all further experiments of this project. Cells were treated for 48 h.

#### Generation of rG-2 cells

CAF-resistant G-2 cells were established by 3 cycles of 3 days chemotherapeutic treatment (1/32, 1/16, 1/16, respectively), each time followed by a recovery time of 10 days with fresh medium.

#### siRNA transfection

Cells were reverse transfected with siRNA using Lipofectamine^®^ RNAiMAX (Thermo Scientific), according to the manufacturer’s instruction. For siRNA sequences (see Table S2).

### In vitro functional assays

#### Proliferation assays

1 × 10^4^ cells per well were seeded on 24-well plates. For EZH2 inhibition experiments, cells were pre-treated for 2 days with different concentrations of EPZ-6438 inhibitor in normal culture medium at the indicated concentration. In experiments assessing resistance to chemotherapy, cells were subsequently treated with a combination of CAF and EPZ-6438 for 2 days, and finally with EPZ-6438 alone for two more days. The cell proliferation was monitored with a Celigo S Cell Imaging Cytometer (Nexcelom, UK). The measurements at different time points were normalized to the respective day 0 values.

#### Crystal violet staining

Cells were stained with crystal violet on the last day of experiments. After a short wash step with PBS, cells were fixed for 2 min with 100% methanol and stained 20 min with 0.05% crystal violet in 20% ethanol. Stained cells were then washed at least three times with tap water and finally dried. Pictures of the staining were taken with an Epson Perfection V700 Photo scanner (Epson).

#### Migration assays

4 × 10^5^ cells were reverse transfected with siRNA or treated with the respective inhibitor in six-wells. On the next day, the adherent cells (~95% confluency) were serum-starved by replacing the complete growth medium with serum-free medium. After 4 h, scratches were performed on the cells monolayer using a sterile pipet tip. The medium was immediately replaced with fresh complete medium. The wounds were photographed at 0 and 24 h and analyzed via ImageJ.

#### Colony formation assay

Cells were pre-treated with inhibitors or transfected with siRNA in the same way as for proliferation assays. The next day, 2 × 10^3^ cells were seeded per well in a six-well plate. Once colonies reached adequate size (generally between 12 and 15 days), cells were fixed and stained as previously described.

### RNA extraction and cDNA synthesis

Total RNA from cultured cells was isolated using Qiazol (Qiagen, Germany) according to the manufacturer’s protocol and quantified using a Denovix DS11 + spectrophotometer (Denovix, USA). 0.5–1 µg of RNA were reverse transcribed using M-MuLV transcriptase and buffer according to the manufacturer protocol (New England Biolabs GmbH, Germany).

### Quantitative real-time PCR (qPCR)

Quantification of gene expression (RT-qPCR) and chromatin occupancy (ChIP-qPCR) were performed in a CFX qPCR-cycler (Bio-Rad, Germany) with 25 µl reaction volumes. The following program was utilized for RT-qPCR: initial denaturation (2 min at 95 °C); 40 cycles of amplification (10 s at 95 °C, 30 s at 60 °C). The housekeeping gene *Rplp0*/*RPLP0* was utilized to normalized gene expression results. For ChIP-qPCR, the following program was used: initial denaturation (2 min at 95 °C); 40 cycles of amplification (15 s at 95 °C, 45 s at 60 °C), termination (1 min at 95 °C, 10 s at 65 °C). The results of chromatin occupancy were normalized to the respective input values and calibrated to their respective controls. Primer sequences utilized in this study are listed in Tables S2 and S3 for RT-qPCR and ChIP-qPCR, respectively.

### Western blotting

Proteins were extracted from six-wells plates with 500 µl ice-cold RIPA buffer (10 mM Tris–Cl pH 8, 1 mM EDTA, 1% v/v Triton X-100, 0.1% sodium deoxycholate, 0.1% SDS, 140 mM NaCl) supplemented + with protease inhibitors cocktail: 1 mM Pefabloc, 1 ng/µl aprotinin/leupeptin, 10 mM BGP, 1 mM NEM and 8 M urea (1/3 of the final volume). Protein samples were then sonicated for five cycles (30 s ON/30 s OFF) in a Bioruptor (Diagenode, Belgium). Samples were then mixed with Lämmli buffer (6×, 375 mM Tris/HCl, 10% SDS, 30% glycerol, 0.02% bromophenol blue, 9.3% DTT) and cooked for 5 min at 95 °C. Same amounts of protein per sample were then separated using 10–12% SDS polyacrylamide gel electrophoresis and transferred onto nitrocellulose membranes (Immobilon, Millipore, USA). Membranes were blocked with 5% skimmed milk in TBS-T and incubated overnight at 4 °C with specific primary antibodies diluted in the same blocking solution. Next, membranes were washed with TBS-T, incubated 1 h with secondary antibodies diluted in blocking buffer, washed again with TBS-T, and finally developed using HRP substrate (Dianova GmbH, Germany) in a Chemidoc imaging system (Biorad, Germany).

### RNA sequencing and analysis

RNA library preparation was carried out using NEXTflex™ Rapid Illumina Directional Kit according to the manufacturer’s instructions. The size of the generated libraries was estimated on a high sensitivity DNA chip (Agilent) using a Bioanalyser 2100 (Agilent) and their concentration was determined using Qubit fluorimeter (Invitrogen). Finally, libraries were multiplexed to a final concentration of 2 nM and sequenced on a HiSeq 2500 Illumina-Sequencer at the NGS-Integrative Genomics (NIG) at the University Medical Center Göttingen (UMG).

The mRNA-seq raw data (Fastq files) were processed in the Galaxy environment (https://galaxy.gwdg.de) provided by the “Gesellschaft für Wissenschaftliche Datenverarbeitung mbH Göttingen” (GWDG) following the pipeline established in the past [78]. After quality check using FastQC, sequencing data were trimmed (FASTQ Trimmer tool), aligned to the murine reference genome (mm9) with the TopHat tool. Next, reads were assigned to their respective genomic features using htseq-count. Finally, differential gene expression analyses were performed using DESeq2. Analyses of gene signature enrichment were performed using the gene set enrichment analysis (GSEA) tool (http://www.broadinstitute.org/gsea/downloads.jsp) and the web-based Enrichr tool (http://amp.pharm.mssm.edu/Enrichr/). The GSEA analyses were ran on a count matrix containing all genes harboring expression level over the background (basemean > 15 normalized counts) with following parameters: Gene set databases Hallmarks (H.all.v7.0), Curated (C2.all.v7.0) and Oncogenic signatures (C6.all.v7.0); collapse = false; permute = gene_set; set_max = 2000; set_min = 15; num = 1000; norm = 200. Heatmap representations of gene expression were generated with the online Morpheus tool (https://software.broadinstitute.org/morpheus/).

Data were deposited at ArrayExpress (www.ebi.ac.uk/arrayexpress/) under the accession number E-MTAB-9547.

### Chromatin immunoprecipitation (ChIP)

Chromatin immunoprecipitation for H3K27me3 and H3K27ac was performed 48 h after chemotherapy or control vehicle treatment, as described previously [79]. Briefly, pG-2 cells were cultured in 15 cm plates. Protein–DNA complexes were crosslinked with 1% formaldehyde (Sigma-Aldrich, Germany) and the nuclear fraction was extracted and sonicated with a Bioruptor pico (Diagenode, Belgium). After controlling the size of the DNA fragments and a pre-cleaning step, the same amounts of samples were incubated with 1 µg anti-H3K27me3 or anti-H3K27ac antibody overnight at 4 °C and immuno-precipitated with protein A-sepharose. Finally, DNA–protein complexes were reverse-crosslinked, DNA fragments were purified by phenol–chloroform extraction, and concentration was determined using Qubit fluorimeter (Invitrogen). Finally, 20–30 µg immunoprecipitated DNA fragments were used for library generation with the MicroPlex Library Preparation Kit v2 (Diagenode). The size of the libraries was estimated with a high sensitivity DNA chip (Agilent) using a Bioanalyser 2100 (Agilent) and libraries were subsequently multiplexed to a final concentration of 2 nM. Sequencing reactions were performed on a HiSeq 4000 Illumina-Sequencer (NGS Integrative Genomics Core Unit, University Medical Center of Göttingen).

Data were deposited at ArrayExpress (www.ebi.ac.uk/arrayexpress/) under the accession number E-MTAB-9584.

### Analysis of ChIP-seq data

ChIP-seq data were processed and analyzed in the Galaxy environment (https://galaxy.gwdg.de/) following a pipeline established in the past [80]. After a quality check (FastQC), reads were aligned to the mouse reference genome (mm9) using Bowtie2. H3K27ac peaks were identified with the MACS2 tool and Differential Binding analyses were performed with Diffbind. The deep tools suite was used for the generation of normalized coverage files (bamCompare). To visualize occupied regions, region scoring matrixes were computed (computeMatrix) and profiles plots or heatmaps were generated (plotProfile and plotHeatmap). Histone modification occupancy at specific genomic regions was visualized with the integrative genome Viewer (IGV; http://software.broadinstitute.org/software/igv/). Enrichments of genomic regions related to specific gene signatures were performed with the Enrichr web-based tool (http://amp.pharm.mssm.edu/Enrichr/).

### IHC staining and scoring

IHC staining was performed as described previously [80]. Briefly, tumor sections were deparaffinized and rehydrated using decreasing alcohol concentration. Antigen retrieval was performed with citric acid buffer (1 mM citric buffer pH 6.0, 0.05% Τween 20) or EDTA buffer (10 mM EDTA, 0.05% Tween 20, pH 8.0) for EZH2 and NFATc1, respectively, in a microwave pressure cooker for 10 min. After blocking endogenous peroxidase and unspecific epitopes, sections were subsequently incubated with primary antibodies overnight at 4 °C, washed with PBT-T, and finally incubated with biotinylated secondary antibodies for 1 h at room temperature. Next, Horse Radish Peroxidase coupled avidin (Sigma-Aldrich, 1:1000 in PBS) was applied for 90 min. The slides were washed in PBS and developed with a DAB-chromogen solution. Counterstain was performed with hematoxylin (Roth). Last, tissues were dehydrated with increasing alcohol concentration and mounted with Histokitt (Roth). Brightfield pictures of the staining were acquired with a Zeiss AXIO Scope.A1 microscope (Zeiss). The list of the utilized antibodies and their dilutions is available in Tables S4 and S5 in the supplemental data. Immunostained WAP-T tumors were scored based on the percentage of DAB-positive cells (EZH2^+^, NFATC1^+^) per acquired field (min. 5 fields per treated group). EZH2 and NFATC1 scoring on the patient tissue-microarrays were established based on the staining intensity (null = no detectable staining, low = weak staining intensity, high = strong staining intensity). Antibodies used for immunohistochemical staining are provided in Supplementary Information.

### Immunofluorescence staining

Cells were plated on glass coverslips and grown in 24-well plates. For immunofluorescence staining, cells were washed with PBS, fixed using 4% paraformaldehyde in PBS for 10 min, and washed again with PBS for 5 min. Cells were then permeabilized with 0.1% Triton X-100 in PBS for 10 min, washed with PBS for 5 min, and blocked 5% BSA in PBS for 30 min. Primary antibodies diluted in blocking solution were applied to the coverslips overnight at 4 °C in a humid chamber. After a washing step in PBS-T, cells were incubated with appropriate fluorophore-conjugated secondary antibodies for 1 h at room temperature in a dark humid chamber. Cells were washed with PBS-T and nuclei were stained with DAPI in PBS (1 µg/ml) for 5 min. After the last wash step in PBS, coverslips were mounted on normal glass slides using Mowiol 4-88 mounting medium (Sigma). Fluorescence pictures were acquired with a Zeiss AXIO Scope.A1 microscope (Zeiss). Detailed lists of primary and secondary antibodies used in this study are provided in the supplemental data. Fluorescence intensity was quantified using the ImageJ software.

### Mouse experiments

All animal experiments were performed according to the German regulations for animal experimentation and authorized by the local ethics office (Niedersächsisches Landesamt für Verbraucherschutz und Lebensmittelsicherheit, LAVES) under the registration number 33.19-42502-04-16/2169.

Mice were housed in a controlled environment at a 12 h dark/light cycle and 22 °C and were fed laboratory chow and tap water ad libitum.

#### Cyclosporin A treatment

Syngeneic virgin female WAP-T mice (Balb/c) were anesthetized with an injection of ketamine/xylazine and 1 × 10^6^ pG-2 cells in 20 µl DMEM were injected in the right abdominal mammary gland. The animals were randomly assigned to four groups: controls (group 1), CsA treatment (group 2), CAF treatment (group 3), and CAF + CsA treatment (group 4). Once tumors reached an average volume of 200 mm², animals of groups 2 and 4 were treated with 5 mg/kg of CsA intraperitoneally (IP), while groups 1 and 3 received equal amounts of the vector solution (2% DMSO; 30% PEG300; 5% Tween 80 in H_2_O) three times a week. On the day following the first CsA injection, animals of groups 3 and 4 were treated with a single dose chemotherapy IP (50 mg/kg cyclophosphamide, 2.5 mg/kg doxorubicin, 50 mg/kg 5-FU), while groups 1 and 2 received a single dose of an equal volume of NaCl 0,9%. Treatment with CsA was continued until the end of the experiment; animal weight and tumor volumes (caliper measurement) were monitored three times a week.

#### Tissues analyzed in IHC staining

The MaCa tissues utilized for IHC staining originated from Jannasch et al. where animals were treated with a single dose combining 100 mg/kg cyclophosphamide, 5 mg/kg doxorubicin, and 100 mg/kg 5-fluorouracil [31].

### Chorio-allantoic membrane (CAM) assay

CAM assays were performed as described previously [81]. Briefly, pG-2 cells were pre-treated with either DMSO, 4 µM CsA, 1/32 CAF or a combination of 4 µM CsA and 1/32 CAF. 48 h later, cells were trypsinized and resuspended in DMEM:Matrigel (1:1) supplemented with DMSO (control and CAF alone groups) or 4 µM CsA (CsA and CsA + CAF groups). For every replicate, 3 × 10^6^ of pretreated pG-2 cells in 40 µl were implanted onto a 10 days old chicken embryo CAM and incubated for further 7 days.

#### Analyzing volumes of the growing tumors via micro-CT

Excised tumors were briefly rinsed five times in water and then transferred to 35% and 70% ethanol (1 h each). For staining and fixation, tumors were placed overnight at room temperature (RT) under slow rotation in a 4% paraformaldehyde solution (PFA, Serva Electrophoresis) in phosphate-buffered saline, pH 7.4 (PBS, Invitrogen), containing 0.7% phosphotungstic acid solution (PTA, Sigma-Aldrich Corp.) diluted in 70% ethanol. Samples were then briefly rinsed in water and stored in fresh 70% ethanol. For further μCT analysis, the PTA-stained tumors were dehydrated with ascending ethanol series and embedded in paraffin (Suesse Labortechnik). The paraffin blocks were scanned in an in vivo microCT system QuantumFX (Perkin Elmer) operated with the following settings: 90 kV tube voltage, 200 µA tube current, 10 × 10 mm^2^ field-of-view, 3 min total acquisition time resulting in 3D data sets with a resolution of ~20 µm. These data sets were visualized and analyzed in Scry7.0 (custom-made render software, Christian Dullin, 2021). A threshold of 12,000 GVal (in the arbitrary units of the CT data sets) was applied to separate tissue from paraffin, air, and the sample holder. A virtual scalpel was utilized to remove residual CAM. Tumor volume was measured by multiplying the number of segmented tumor voxels with the voxel volume.

### Statistical analysis

Statistical analyses were performed with GraphPad Prism version 8.0.1. Appropriate statistical tests were used for quantitative PCR, densitometry, functional assays (colony number, confluency, population, migration, and scratch assays), immunohistochemistry scoring, fluorescence intensity and survival analyses results and mentioned at the respective figure legends (*p < 0.05, **p < 0.01, ***p < 0.001).

### Reporting summary

Further information on research design is available in the Nature Research Reporting Summary linked to this article.

## Supplementary information

<b>Authorship agreement</b>

<b>Supplemental data - clean</b>

<b>Reporting Summary</b>

<b>Figure S1</b>

<b>Figure S2</b>

<b>Figure S3</b>

<b>Figure S4</b>

<b>Figure S5</b>

<b>Figure S6</b>

## Data Availability

High throughput sequencing datasets generated and analyzed during the current study are available in the ArrayExpress repository (https://www.ebi.ac.uk/arrayexpress/) under the accession numbers E-MTAB-9584 and E-MTAB-9547. Publicly available data analyzed during the current study are available in the MERAV (http://merav.wi.mit.edu), the R2: Genomics Analysis and Visualization Platform (https://hgserver1.amc.nl/cgi-bin/r2/main.cgi) and the Xena browser (https://xenabrowser.net/) repositories.

## References

[1] Ferlay J, Ervik M, Lam F, Colombet M, Mery L, Piñeros M, et al. Global Cancer Observatory: Cancer Today. Lyon, France: International Agency for Research on Cancer. [Internet]. https://gco.iarc.fr/today. Accessed 29 Sept 2021.

[2] Gucalp A, Traina TA, Eisner JR, Parker JS, Selitsky SR, Park BH (2019). Male breast cancer: a disease distinct from female breast cancer. Breast Cancer Res Treat.

[3] Harbeck N, Gnant M (2017). Breast cancer. Lancet.

[4] Veronesi U, Cascinelli N, Mariani L, Greco M, Saccozzi R, Luini A (2002). Twenty-year follow-up of a randomized study comparing breast-conserving surgery with radical mastectomy for early breast cancer. N Engl J Med.

[5] Fisher B, Anderson S, Bryant J, Margolese RG, Deutsch M, Fisher ER (2002). Twenty-year follow-up of a randomized trial comparing total mastectomy, lumpectomy, and lumpectomy plus irradiation for the treatment of invasive breast cancer. N Engl J Med.

[6] Colzani E, Johansson ALVV, Liljegren A, Foukakis T, Clements M, Adolfsson J (2014). Time-dependent risk of developing distant metastasis in breast cancer patients according to treatment, age and tumour characteristics. Br J Cancer.

[7] Mathiesen RR, Fjelldal R, Liestøl K, Due EU, Geigl JB, Riethdorf S (2012). High-resolution analyses of copy number changes in disseminated tumor cells of patients with breast cancer. Int J Cancer.

[8] Prat A, Pineda E, Adamo B, Galván P, Fernández A, Gaba L (2015). Clinical implications of the intrinsic molecular subtypes of breast cancer. Breast.

[9] Perou CM, Sørile T, Eisen MB, Van De Rijn M, Jeffrey SS, Ress CA (2000). Molecular portraits of human breast tumours. Nature.

[10] Prat A, Fan C, Fernández A, Hoadley KA, Martinello R, Vidal M (2015). Response and survival of breast cancer intrinsic subtypes following multi-agent neoadjuvant chemotherapy. BMC Med.

[11] Lu W, Kang Y (2019). Epithelial–mesenchymal plasticity in cancer progression and metastasis. Dev Cell.

[12] Ye X, Weinberg RA. Epithelial–mesenchymal plasticity: a central regulator of cancer progression. vol. 25, Trends in cell biology. Elsevier Ltd; 2015. p. 675–86. 10.1016/j.tcb.2015.07.012 PMC4628843 26437589

[13] Mohammad HP, Barbash O, Creasy CL (2019). Targeting epigenetic modifications in cancer therapy: erasing the roadmap to cancer. Nat Med.

[14] Wainwright EN, Scaffidi P (2017). Epigenetics and cancer stem cells: unleashing, hijacking, and restricting cellular plasticity. Trends Cancer.

[15] Skrypek N, Goossens S, De Smedt E, Vandamme N, Berx G (2017). Epithelial-to-mesenchymal transition: epigenetic reprogramming driving cellular plasticity. Trends Genet.

[16] Antonysamy S, Condon B, Druzina Z, Bonanno JB, Gheyi T, Zhang F (2013). Structural context of disease-associated mutations and putative mechanism of autoinhibition revealed by X-Ray crystallographic analysis of the EZH2-SET domain. PLoS ONE.

[17] Simon JA, Kingston RE. Occupying chromatin: polycomb mechanisms for getting to genomic targets, stopping transcriptional traffic, and staying put. Mol Cell. 2013;49:808–24. 10.1016/j.molcel.2013.02.013 PMC3628831 23473600

[18] Vizán P, Beringer M, Ballaré C, Di Croce L (2015). Role of PRC2-associated factors in stem cells and disease. FEBS J.

[19] Margueron R, Reinberg D (2011). The Polycomb complex PRC2 and its mark in life. Nature.

[20] Wen Y, Cai J, Hou Y, Huang Z, Wang Z (2017). Role of EZH2 in cancer stem cells: from biological insight to a therapeutic target. Oncotarget.

[21] Herranz N, Pasini D, Díaz VM, Francí C, Gutierrez A, Dave N (2008). Polycomb complex 2 Is required for E-cadherin repression by the Snail1 transcription factor. Mol Cell Biol.

[22] Martínez-Fernández M, Dueñas M, Feber A, Segovia C, García-Escudero R, Rubio C (2015). A Polycomb-mir200 loop regulates clinical outcome in bladder cancer. Oncotarget.

[23] Schulze-Garg C, Löhler J, Gocht A, Deppert W (2000). A transgenic mouse model for the ductal carcinoma in situ (DCIS) of the mammary gland. Oncogene.

[24] Maenz C, Lenfert E, Pantel K, Schumacher U, Deppert W, Wegwitz F (2015). Epithelial-mesenchymal plasticity is a decisive feature for the metastatic outgrowth of disseminated WAP-T mouse mammary carcinoma cells. BMC Cancer.

[25] Otto B, Gruner K, Heinlein C, Wegwitz F, Nollau P, Ylstra B (2013). Low-grade and high-grade mammary carcinomas in WAP-T transgenic mice are independent entities distinguished by Met expression. Int J Cancer.

[26] Lenfert E, Maenz C, Heinlein C, Jannasch K, Schumacher U, Pantel K (2015). Mutant p53 promotes epithelial–mesenchymal plasticity and enhances metastasis in mammary carcinomas of WAP-T mice. Int J Cancer.

[27] Wegwitz F, Lenfert E, Gerstel D, von Ehrenstein L, Einhoff J, Schmidt G (2016). CEACAM1 controls the EMT switch in murine mammary carcinoma in vitro and in vivo. Oncotarget.

[28] Gerstel D, Wegwitz F, Jannasch K, Ludewig P, Scheike K, Alves F (2011). CEACAM1 creates a pro-angiogenic tumor microenvironment that supports tumor vessel maturation. Oncogene.

[29] Wegwitz F, Kluth M-AA, Mänz C, Otto B, Gruner K, Heinlein C, et al. Tumorigenic WAP-T mouse mammary carcinoma cells: a model for a self-reproducing homeostatic cancer cell system. Najbauer J, editor. PLoS ONE 2010;5:e12103. 10.1371/journal.pone.0012103 PMC2920333 20730114

[30] Quante T, Wegwitz F, Abe J, Rossi A, Deppert W, Bohn W. Aberrant proliferation of differentiating alveolar cells induces hyperplasia in resting mammary glands of SV40-TAg transgenic mice. Front Oncol. 2014;4:168. 10.3389/fonc.2014.00168 PMC4071642 25019062

[31] Jannasch K, Wegwitz F, Lenfert E, Maenz C, Deppert W, Alves F (2015). Chemotherapy of WAP-T mouse mammary carcinomas aggravates tumor phenotype and enhances tumor cell dissemination. Int J Cancer.

[32] Kiesslich T, Pichler M, Neureiter D (2012). Epigenetic control of epithelial–mesenchymal-transition in human cancer. Mol Clin Oncol.

[33] Cardenas H, Zhao J, Vieth E, Nephew KP, Matei D (2016). EZH2 inhibition promotes epithelial-to-mesenchymal transition in ovarian cancer cells. Oncotarget.

[34] Song X, Gao T, Wang N, Feng Q, You X, Ye T (2016). Selective inhibition of EZH2 by ZLD1039 blocks H3K27methylation and leads to potent anti-tumor activity in breast cancer. Sci Rep.

[35] Gonzalez ME, Li X, Toy K, DuPrie M, Ventura AC, Banerjee M (2009). Downregulation of EZH2 decreases growth of estrogen receptor-negative invasive breast carcinoma and requires BRCA1. Oncogene.

[36] Chen NM, Neesse A, Dyck ML, Steuber B, Koenig AO, Lubeseder-Martellato C (2017). Context-dependent epigenetic regulation of nuclear factor of activated T cells 1 in pancreatic plasticity. Gastroenterology.

[37] Flores C, Fouquet G, Moura IC, Maciel TT, Hermine O (2019). Lessons to learn from low-dose cyclosporin-A: a new approach for unexpected clinical applications. Front Immunol.

[38] Sengupta S, Jana S, Biswas S, Mandal PK, Bhattacharyya A (2013). Cooperative involvement of NFAT and SnoN mediates transforming growth factor-β (TGF-β) induced EMT in metastatic breast cancer (MDA-MB 231) cells. Clin Exp Metastasis.

[39] Singh SK, Chen N, Hessmann E, Siveke J, Lahmann M, Singh G (2015). Antithetical NFAT c1–Sox2 and p53–miR200 signaling networks govern pancreatic cancer cell plasticity. EMBO J.

[40] Steinbichler TB, Dudás J, Skvortsov S, Ganswindt U, Riechelmann H, Skvortsova II (2018). Therapy resistance mediated by cancer stem cells. Semin Cancer Biol.

[41] Creighton CJ, Li X, Landis M, Dixon JM, Neumeister VM, Sjolund A (2009). Residual breast cancers after conventional therapy display mesenchymal as well as tumor-initiating features. Proc Natl Acad Sci USA.

[42] Scheel C, Weinberg RA (2011). Phenotypic plasticity and epithelial-mesenchymal transitions in cancer and normal stem cells?. Int J Cancer.

[43] Kalluri R, Weinberg RA. The basics of epithelial–mesenchymal transition. J Clin Investig 2009;119:1420–8. 10.1172/JCI39104 PMC2689101 19487818

[44] Mani SA, Guo W, Liao MJ, Eaton EN, Ayyanan A, Zhou AY (2008). The epithelial–mesenchymal transition generates cells with properties of stem cells. Cell.

[45] Hennessy BT, Gonzalez-Angulo AM, Stemke-Hale K, Gilcrease MZ, Krishnamurthy S, Lee JS (2009). Characterization of a naturally occurring breast cancer subset enriched in epithelial-to-mesenchymal transition and stem cell characteristics. Cancer Res.

[46] Loret N, Denys H, Tummers P, Berx G (2019). The role of epithelial-to-mesenchymal plasticity in ovarian cancer progression and therapy resistance. Cancers.

[47] Gooding AJ, Schiemann WP (2020). Epithelial–mesenchymal transition programs and cancer stem cell phenotypes: mediators of breast cancer therapy resistance. Mol Cancer Res.

[48] Guo L, Lee YT, Zhou Y, Huang Y. Targeting epigenetic regulatory machinery to overcome cancer therapy resistance. Semin Cancer Biol. 2021:S1044-579X(20)30282-0. 10.1016/j.semcancer.2020.12.022 PMC8257754 33421619

[49] Deblois G, Madani Tonekaboni SA, Grillo G, Martinez C, Kao YI, Tai F, et al. Epigenetic switch-induced viral mimicry evasion in chemotherapy resistant breast cancer. Cancer Discov. 2020;10:1312–1329. 10.1158/2159-8290.CD-19-1493 32546577

[50] Yomtoubian S, Lee SB, Verma A, Izzo F, Markowitz G, Choi H (2020). Inhibition of EZH2 catalytic activity selectively targets a metastatic subpopulation in triple-negative breast cancer. Cell Rep.

[51] Zhang Y, Liu G, Lin C, Liao G, Tang B (2013). Silencing the EZH2 gene by RNA interference reverses the drug resistance of human hepatic multidrug-resistant cancer cells to 5-Fu. Life Sci.

[52] Hu S, Yu L, Li Z, Shen Y, Wang J, Cai J (2010). Overexpression of EZH2 contributes to acquired cisplatin resistance in ovarian cancer cells in vitro and in vivo. Cancer Biol Ther.

[53] Chien Y-C, Liu L-C, Ye H-Y, Wu J-Y, Yu Y-L (2018). EZH2 promotes migration and invasion of triple-negative breast cancer cells via regulating TIMP2-MMP-2/-9 pathway. Am J Cancer Res.

[54] Huang JP, Ling K (2017). EZH2 and histone deacetylase inhibitors induce apoptosis in triple negative breast cancer cells by differentially increasing H3 Lys27 acetylation in the BIM gene promoter and enhancers. Oncol Lett.

[55] Adelaiye-Ogala R, Budka J, Damayanti NP, Arrington J, Ferris M, Hsu C-C (2017). EZH2 modifies sunitinib resistance in renal cell carcinoma by kinome reprogramming. Cancer Res.

[56] Li Z, Hou P, Fan D, Dong M, Ma M, Li H (2017). The degradation of EZH2 mediated by lncRNA ANCR attenuated the invasion and metastasis of breast cancer. Cell Death Differ.

[57] Hirukawa A, Smith HW, Zuo D, Dufour CR, Savage P, Bertos N (2018). Targeting EZH2 reactivates a breast cancer subtype-specific anti-metastatic transcriptional program. Nat Commun.

[58] Labbé DP, Sweeney CJ, Brown M, Galbo P, Rosario S, Wadosky KM (2017). TOP2A and EZH2 provide early detection of an aggressive prostate cancer subgroup. Clin Cancer Res.

[59] Kikuchi J, Koyama D, Wada T, Izumi T, Hofgaard PO, Bogen B (2015). Phosphorylation-mediated EZH2 inactivation promotes drug resistance in multiple myeloma. J Clin Investig.

[60] Ning X, Shi Z, Liu X, Zhang A, Han L, Jiang K (2015). DNMT1 and EZH2 mediated methylation silences the microRNA-200b/a/429 gene and promotes tumor progression. Cancer Lett.

[61] Vo BHT, Li C, Morgan MA, Theurillat I, Finkelstein D, Wright S (2017). Inactivation of Ezh2 upregulates Gfi1 and drives aggressive Myc-driven Group 3 medulloblastoma. Cell Rep.

[62] Wassef M, Rodilla V, Teissandier A, Zeitouni B, Gruel N, Sadacca B (2015). Impaired PRC2 activity promotes transcriptional instability and favors breast tumorigenesis. Genes Dev.

[63] Sashida G, Wang C, Tomioka T, Oshima M, Aoyama K, Kanai A (2016). The loss of Ezh2 drives the pathogenesis of myelofibrosis and sensitizes tumor-initiating cells to bromodomain inhibition. J Exp Med.

[64] Serresi M, Siteur B, Hulsman D, Company C, Schmitt MJ, Lieftink C (2018). Ezh2 inhibition in Kras-driven lung cancer amplifies inflammation and associated vulnerabilities. J Exp Med.

[65] Serresi M, Gargiulo G, Proost N, Siteur B, Cesaroni M, Koppens M (2016). Polycomb repressive complex 2 is a barrier to KRAS-driven inflammation and epithelial–mesenchymal transition in non-small-cell lung cancer. Cancer Cell.

[66] Wang Y, Hou N, Cheng X, Zhang J, Tan X, Zhang C (2017). Ezh2 acts as a tumor suppressor in kras-driven lung adenocarcinoma. Int J Biol Sci.

[67] Ariës IM, Bodaar K, Karim SA, Chonghaile TN, Hinze L, Burns MA, et al. PRC2 loss induces chemoresistance by repressing apoptosis in T cell acute lymphoblastic leukemia. J Exp Med. 2018;215:3094–3114. 10.1084/jem.20180570 PMC6279404 30404791

[68] Göllner S, Oellerich T, Agrawal-Singh S, Schenk T, Klein HU, Rohde C (2017). Loss of the histone methyltransferase EZH2 induces resistance to multiple drugs in acute myeloid leukemia. Nat Med.

[69] Quan C, Chen Y, Wang X, Yang D, Wang Q, Huang Y (2020). Loss of histone lysine methyltransferase EZH2 confers resistance to tyrosine kinase inhibitors in non-small cell lung cancer. Cancer Lett.

[70] Wang Q, Chen X, Jiang Y, Liu S, Liu H, Sun X, et al. Elevating H3K27me3 level sensitizes colorectal cancer to oxaliplatin. J Mol Cell Biol. 2020;12:125–137. 10.1093/jmcb/mjz032 PMC7109602 31065671

[71] Liu F, Zhu Z, Mao Y, Liu M, Tang T, Qiu S (2009). Inhibition of titanium particle-induced osteoclastogenesis through inactivation of NFATc1 by VIVIT peptide. Biomaterials.

[72] Aramburu J, Yaffe MB, López-Rodríguez C, Cantley LC, Hogan PG, Rao A (1999). Affinity-driven peptide selection of an NFAT inhibitor more selective than cyclosporin A. Science (80-).

[73] Tran Quang C, Leboucher S, Passaro D, Fuhrmann L, Nourieh M, Vincent-Salomon A (2015). The calcineurin/NFAT pathway is activated in diagnostic breast cancer cases and is essential to survival and metastasis of mammary cancer cells. Cell Death Dis.

[74] Im JY, Lee KW, Won KJ, Kim BK, Ban HS, Yoon SH (2016). DNA damage-induced apoptosis suppressor (DDIAS), a novel target of NFATc1, is associated with cisplatin resistance in lung cancer. Biochim Biophys Acta - Mol Cell Res.

[75] Metzelder SK, Michel C, von Bonin M, Rehberger M, Hessmann E, Inselmann S (2015). NFATc1 as a therapeutic target in FLT3-ITD-positive AML. Leukemia.

[76] Kawahara T, Kashiwagi E, Ide H, Li Y, Zheng Y, Miyamoto Y (2015). Cyclosporine A and tacrolimus inhibit bladder cancer growth through down-regulation of NFATc1. Oncotarget.

[77] Klein-Hessling S, Muhammad K, Klein M, Pusch T, Rudolf R, Flöter J, et al. NFATc1 controls the cytotoxicity of CD8+ T cells. Nat Commun. 2017;8:511. 10.1038/s41467-017-00612-6 PMC5593830 28894104

[78] Prokakis E, Dyas A, Grün R, Fritzsche S, Bedi U, Kazerouni ZB (2021). USP22 promotes HER2-driven mammary carcinoma aggressiveness by suppressing the unfolded protein response. Oncogene.

[79] Hamdan FH, Johnsen SA (2018). DeltaNp63-dependent super enhancers define molecular identity in pancreatic cancer by an interconnected transcription factor network. Proc Natl Acad Sci USA.

[80] Wegwitz F, Prokakis E, Pejkovska A, Kosinsky RL, Glatzel M, Pantel K, et al. The histone H2B ubiquitin ligase RNF40 is required for HER2-driven mammary tumorigenesis. Cell Death Dis. 2020;11:873. 10.1038/s41419-020-03081-w PMC7568723 33070155

[81] Wilting J, Christ B, Bokeloh M (1991). A modified chorioallantoic membrane (CAM) assay for qualitative and quantitative study of growth factors—studies on the effects of carriers, PBS, angiogenin, and bFGF. Anat Embryol.

