# Supplementary material for: Decreased PRC2 activity supports the survival of basal-like breast cancer cells to cytotoxic treatments
Source: Cell Death Dis. 2021 Nov 29;12(12):1118. doi: 10.1038/s41419-021-04407-y (PMC8630036; doi:10.1038/s41419-021-04407-y)
Supplement: Supplementary file 2 — Supplemental data - clean [file 41419_2021_4407_MOESM2_ESM.docx]

**SUPPLEMENTAL DATA**

**Supplemental Figures Legends:**

**Figure S1: WAP-T cells surviving a CAF treatment gain stem cell and EMT properties *in vitro*.
A:** Phase contrast pictures of resistant G-2 cells (rG-2) treated or not with CAF for 48 hours. Scale bar = 250 µm **B:** Crystal violet staining showing that rG-2 cells tolerate higher CAF doses than their parental counterpart. **C:** When compared to the parental cell line, rG-2 cells show enhanced EMT- and CSC-associated gene expression signatures under normal culture conditions, as assessed by RT-qPCR. CAF-treated rG-2 cells maintain these characteristics. Statistical tests = one-way ANOVA. All experiments were performed in biological triplicates. Error bars: standard deviation of the mean (SEM). * p-val<0.05, ** p-val<0.01, *** p-val<0.005.

**Figure S2: WAP-T tumor cells surviving CAF-treatment downregulate the expression of PRC2 core subunits.
A:** pG-2 cells treated with CAF for 48h enrich for gene signatures characteristic for PRC2 loss, as identified by mRNA-seq followed by GSEA. **B:** rG-2 cells show significantly reduced expression of four core subunits of the PRC2 complex (*Ezh2*, *Suz12*, *Rbbp7,* and *Mtf2)* under basal growth conditions, as assessed by RT-qPCR. The expression of these genes was even stronger reduced upon 48h CAF treatment. Statistical tests = one-way ANOVA. **C**: Western blot showing a reduction of SUZ12 in pG-2 cells upon CAF treatment **D**: Densitometry analysis showing a reduction of the EZH2 protein levels in CAF treated pG-2 cells. Student t-test. All experiments were performed in biological triplicates. Error bars: standard error of the mean (SEM). *** p-val<0.005.

**Figure S3: Reduction of EZH2 activity enhance the aggressiveness of several human tumor cell lines.
A** and **B**: Effects of EZH2 knockdown on the growth kinetics of the human BLBC cell line MDA-BM-468 treated (**B**) or not (**A**) with CAF for 48h, measured with a Celigo® and by crystal violet staining. **C to F**: Treatment of human cancer cell lines of various origins with increasing concentrations of the EZH2 inhibitor EPZ-6438 strongly enhances their proliferative properties, as assessed with a Celigo®. Student t-test: **A-B**. All experiments were performed in biological triplicates. Error bars: standard error of the mean (SEM). *** p-val<0.005, ns: not significant.

**Figure S4: Reduction of PRC2 activity during chemotherapy treatment allows activation gene expression programs promoting tumor cell survival**.
**A** and **B**: ChIP-seq data showing a gain of H3K27ac occupancy (**A**) and a loss of H3K27me3 (**B**) upon 48h CAF treatment of pG-2 cells. The H3K27me3 and H3K27ac occupancy is provided for all regions occupied in the control and/or CAF treated state, genome-wide. Changes of occupancy are shown as plot profiles (left panel) and as a heatmap (middle panel). Changes of H3K27ac (**A**) and H3K27me3 (**B**) occupancy were quantified and are provided as a violin plot in the respective right panels. Statistical test: Mann Witney. **C**: ChIP-seq tracks exemplary showing the epigenetic switch from transcriptional repressive H3K27me3 to activating H3K27ac mark occurring at the *Hgma2* and *Fgfr2* locus upon 48h CAF treatment. **D**: ChIP-qPCR validating H3K27me3 loss and H3K27ac gain at the *Hmga2* promoter-proximal region upon 48h CAF treatment. Data were normalized to their respective input. The dashed line represents the background signal obtained in the IgG group. **E**: RT-qPCR showing an increase of *Fgfr2* and *Hgma2* gene expression upon CAF treatment. The data were normalized to the housekeeping gene *Rplp0* and calibrated to the vehicle (Veh) condition. **F**: Densitometry analysis of the NFATc1 levels in pG-2 cells w/ or w/o CAF treatment. The densitometry values represent the normalized NFATc1/Actin signal. **D-F**: Student t-test. All experiments were performed in biological triplicates. Error bars: standard deviation of the mean (SEM). * p-val<0.05

**Figure S5: Loss of EZH2 stimulates NFATc1 expression and correlates with poor breast cancer patient prognosis.
A** and **B**: Treatment of pG-2 (**A**) and rG-2 (**B**) cells with the EZH2 inhibitor EPZ-6438 (250 nM, 96h) increases the NFATc1 protein levels, as assessed by western blot. **C** and **D**: Treatment of MDA-MB-468 cells with EPZ-6438 (500 nM, 96h) increases NFATc1 expression at the mRNA and protein level, as assessed via RT-qPCR (**C**) and western blot (**D**), respectively. n = 3 biological replicates. RT-qPCR data were normalized to *Rplp0* and calibrated to the vehicle (veh) condition. Error bars = SEM, statistic = student t-test (**C**). The densitometry values provide the normalized signals NFATc1/Actin or H3K27me3/H3, respectively (**A, B and D**). **E**: Overexpression of EZH2 in pG-2 cells lead to a significant reduction of Nfatc1 levels as assessed via RT-qPCR. EV: empty vector, OE: overexpression. Student t-test. **F**: Quantification of NFATc1 and EZH2 positive cells in immunostainings of murine tumors from groups 1, 2 and 3. Fisher’s exact test **G**: Representative pictures of EZH2 and NFATc1 staining in a breast cancer TMA. **H and I**: ROC analyses on publically available TNBC data (<http://www.rocplot.org/>) demonstrate that patients showing poor response to chemotherapy express lower *EZH2*- (**H**) and higher *NFATc1* (**I**) levels. Box plots: Mann Whitney test. * p-val<0.05, ** p-val<0.01, *** p-val<0.005.

**Figure S6: Inhibition of NFATc1 decreases TNBC cells aggressiveness
A:** Validation of the NFATc1 knockdown efficiency in MDA-MB-468 via western blot. **B**: Assessment of MDA-MB-468 growth kinetics upon NFATc1 knockdown with a Celigo®. **C** and **D**: Effects of Cyclosporin A (CsA) (**C**) or VIVIT (**D**) on pG-2 cell proliferation *in vitro*. Growth kinetics were assessed by Celigo® confluence measurement (left panel) and crystal violet staining (right panel). Error bars depicted as shadow area: SEM. **E**: Treatment of MDA-MB-468 cells with increasing concentrations of cyclosporine A (CsA, 6 days) impairs their growth properties and sensitizes cells to CAF therapy (48h), as assessed by crystal violet staining (lower panel) and subsequently quantified (upper panel). **C-E**: statistics = one-way ANOVA. All experiments were performed in biological triplicates. Error bars: standard deviation of the mean (SEM). ** p-val<0.01, *** p-val<0.005.

**Supplemental Material**

**Supplemental Tables**

**Table S1 Epigenetic regulators list (361 genes)**

| **Gene_name** | **Gene_ID** | **Gene_description** |
| --- | --- | --- |
| AIRE | ENSG00000160224 | autoimmune regulator |
| AOF2 | ENSG00000004487 | Amine Oxidase (Flavin Containing) Domain 2 |
| ARID1A | ENSG00000117713 | AT-rich interaction domain 1A |
| ARID4A | ENSG00000032219 | AT-rich interaction domain 4A |
| ARID4B | ENSG00000054267 | AT-rich interaction domain 4B |
| ASF1A | ENSG00000111875 | anti-silencing function 1A histone chaperone |
| ASF1B | ENSG00000105011 | anti-silencing function 1B histone chaperone |
| ASH1L | ENSG00000116539 | ASH1 like histone lysine methyltransferase |
| ASH2L | ENSG00000129691 | ASH2 like, histone lysine methyltransferase complex subunit |
| ASXL1 | ENSG00000171456 | ASXL transcriptional regulator 1 |
| ASXL2 | ENSG00000143970 | ASXL transcriptional regulator 2 |
| ASXL3 | ENSG00000141431 | ASXL transcriptional regulator 3 |
| ATAD2 | ENSG00000156802 | ATPase family AAA domain containing 2 |
| ATAD2B | ENSG00000119778 | ATPase family AAA domain containing 2B |
| ATRX | ENSG00000085224 | ATRX chromatin remodeler |
| ATXN7 | ENSG00000163635 | ataxin 7 |
| ATXN7L3 | ENSG00000087152 | ataxin 7 like 3 |
| BAZ1A | ENSG00000198604 | bromodomain adjacent to zinc finger domain 1A |
| BAZ1B | ENSG00000009954 | bromodomain adjacent to zinc finger domain 1B |
| BAZ2A | ENSG00000076108 | bromodomain adjacent to zinc finger domain 2A |
| BAZ2B | ENSG00000123636 | bromodomain adjacent to zinc finger domain 2B |
| BMI1 | ENSG00000168283 | BMI1 proto-oncogene, polycomb ring finger |
| BPTF | ENSG00000171634 | bromodomain PHD finger transcription factor |
| BRD1 | ENSG00000100425 | bromodomain containing 1 |
| BRD2 | ENSG00000204256 | bromodomain containing 2 |
| BRD3 | ENSG00000169925 | bromodomain containing 3 |
| BRD4 | ENSG00000141867 | bromodomain containing 4 |
| BRD7 | ENSG00000166164 | bromodomain containing 7 |
| BRD8 | ENSG00000112983 | bromodomain containing 8 |
| BRD9 | ENSG00000028310 | bromodomain containing 9 |
| BRDT | ENSG00000137948 | bromodomain testis associated |
| BRMS1 | ENSG00000174744 | BRMS1 transcriptional repressor and anoikis regulator |
| BRMS1L | ENSG00000100916 | BRMS1 like transcriptional repressor |
| BRPF1 | ENSG00000156983 | bromodomain and PHD finger containing 1 |
| BRPF3 | ENSG00000096070 | bromodomain and PHD finger containing 3 |
| BRWD1 | ENSG00000185658 | bromodomain and WD repeat domain containing 1 |
| BRWD3 | ENSG00000165288 | bromodomain and WD repeat domain containing 3 |
| RIOX1 | ENSG00000170468 | Ribosomal Oxygenase 1 |
| CARM1 | ENSG00000142453 | coactivator associated arginine methyltransferase 1 |
| CBX1 | ENSG00000108468 | chromobox 1 |
| CBX2 | ENSG00000173894 | chromobox 2 |
| CBX3 | ENSG00000122565 | chromobox 3 |
| CBX4 | ENSG00000141582 | chromobox 4 |
| CBX5 | ENSG00000094916 | chromobox 5 |
| CBX6 | ENSG00000183741 | chromobox 6 |
| CBX7 | ENSG00000100307 | chromobox 7 |
| CBX8 | ENSG00000141570 | chromobox 8 |
| CCDC101 | ENSG00000176476 | Coiled-Coil Domain-Containing Protein 101 |
| CDC73 | ENSG00000134371 | cell division cycle 73 |
| CDY1 | ENSG00000172288 | chromodomain Y-linked 1 |
| CDY1B | ENSG00000172352 | chromodomain Y-linked 1B |
| CDY2A | ENSG00000182415 | chromodomain Y-linked 2A |
| CDY2B | ENSG00000129873 | chromodomain Y-linked 2B |
| CDYL | ENSG00000153046 | chromodomain Y like |
| CDYL2 | ENSG00000166446 | chromodomain Y like 2 |
| CECR2 | ENSG00000099954 | CECR2 histone acetyl-lysine reader |
| CHAF1A | ENSG00000167670 | chromatin assembly factor 1 subunit A |
| CHAF1B | ENSG00000159259 | chromatin assembly factor 1 subunit B |
| CHD1 | ENSG00000153922 | chromodomain helicase DNA binding protein 1 |
| CHD1L | ENSG00000131778 | chromodomain helicase DNA binding protein 1 like |
| CHD2 | ENSG00000173575 | chromodomain helicase DNA binding protein 2 |
| CHD3 | ENSG00000170004 | chromodomain helicase DNA binding protein 3 |
| CHD4 | ENSG00000111642 | chromodomain helicase DNA binding protein 4 |
| CHD5 | ENSG00000116254 | chromodomain helicase DNA binding protein 5 |
| CHD6 | ENSG00000124177 | chromodomain helicase DNA binding protein 6 |
| CHD7 | ENSG00000171316 | chromodomain helicase DNA binding protein 7 |
| CHD8 | ENSG00000100888 | chromodomain helicase DNA binding protein 8 |
| CHD9 | ENSG00000177200 | chromodomain helicase DNA binding protein 9 |
| CREBBP | ENSG00000005339 | CREB binding protein |
| CTR9 | ENSG00000198730 | CTR9 homolog, Paf1/RNA polymerase II complex component |
| CXXC1 | ENSG00000154832 | CXXC finger protein 1 |
| DIDO1 | ENSG00000101191 | death inducer-obliterator 1 |
| DMAP1 | ENSG00000178028 | DNA methyltransferase 1 associated protein 1 |
| DNAPTP3 |  | DNA polymerase-transactivated protein 3 |
| DNMT1 | ENSG00000130816 | DNA methyltransferase 1 |
| DNMT3A | ENSG00000119772 | DNA methyltransferase 3 alpha |
| DNMT3B | ENSG00000088305 | DNA methyltransferase 3 beta |
| DNMT3L | ENSG00000142182 | DNA methyltransferase 3 like |
| DOT1L | ENSG00000104885 | DOT1 like histone lysine methyltransferase |
| DPF1 | ENSG00000011332 | double PHD fingers 1 |
| DPF2 | ENSG00000133884 | double PHD fingers 2 |
| DPF3 | ENSG00000205683 | double PHD fingers 3 |
| DPY30 | ENSG00000162961 | dpy-30 histone methyltransferase complex regulatory subunit |
| DZIP3 | ENSG00000198919 | DAZ interacting zinc finger protein 3 |
| EED | ENSG00000074266 | embryonic ectoderm development |
| EHMT1 | ENSG00000181090 | euchromatic histone lysine methyltransferase 1 |
| EHMT2 | ENSG00000204371 | euchromatic histone lysine methyltransferase 2 |
| ELP3 | ENSG00000134014 | elongator acetyltransferase complex subunit 3 |
| EP300 | ENSG00000100393 | E1A binding protein p300 |
| EP400 | ENSG00000183495 | E1A binding protein p400 |
| EPC1 | ENSG00000120616 | enhancer of polycomb homolog 1 |
| EPC2 | ENSG00000135999 | enhancer of polycomb homolog 2 |
| EZH1 | ENSG00000108799 | enhancer of zeste 1 polycomb repressive complex 2 subunit |
| EZH2 | ENSG00000106462 | enhancer of zeste 2 polycomb repressive complex 2 subunit |
| FANCL | ENSG00000115392 | FA complementation group L |
| FBXO10 | ENSG00000147912 | F-box protein 10 |
| FBXO11 | ENSG00000138081 | F-box protein 11 |
| G2E3 | ENSG00000092140 | G2/M-phase specific E3 ubiquitin protein ligase |
| GADD45A | ENSG00000116717 | growth arrest and DNA damage inducible alpha |
| HAT1 | ENSG00000128708 | histone acetyltransferase 1 |
| HCFC1 | ENSG00000172534 | host cell factor C1 |
| HDAC1 | ENSG00000116478 | histone deacetylase 1 |
| HDAC10 | ENSG00000100429 | histone deacetylase 10 |
| HDAC11 | ENSG00000163517 | histone deacetylase 11 |
| HDAC2 | ENSG00000196591 | histone deacetylase 2 |
| HDAC3 | ENSG00000171720 | histone deacetylase 3 |
| HDAC4 | ENSG00000068024 | histone deacetylase 4 |
| HDAC5 | ENSG00000108840 | histone deacetylase 5 |
| HDAC6 | ENSG00000094631 | histone deacetylase 6 |
| HDAC7 | ENSG00000061273 | histone deacetylase 7 |
| HDAC8 | ENSG00000147099 | histone deacetylase 8 |
| HDAC9 | ENSG00000048052 | histone deacetylase 9 |
| HIRA | ENSG00000100084 | histone cell cycle regulator |
| HLTF | ENSG00000071794 | helicase like transcription factor |
| HTATIP | ENSG00000172977 | HIV-1 Tat Interactive Protein, 60kDa |
| ING1 | ENSG00000153487 | inhibitor of growth family member 1 |
| ING2 | ENSG00000168556 | inhibitor of growth family member 2 |
| ING3 | ENSG00000071243 | inhibitor of growth family member 3 |
| ING4 | ENSG00000111653 | inhibitor of growth family member 4 |
| ING5 | ENSG00000168395 | inhibitor of growth family member 5 |
| INTS12 | ENSG00000138785 | integrator complex subunit 12 |
| JARID2 | ENSG00000008083 | jumonji and AT-rich interaction domain containing 2 |
| JHDM1D | ENSG00000006459 | Jumonji C Domain Containing Histone Demethylase 1 Homolog D |
| JMJD1C | ENSG00000171988 | jumonji domain containing 1C |
| JMJD4 | ENSG00000081692 | jumonji domain containing 4 |
| JMJD6 | ENSG00000070495 | jumonji domain containing 6, arginine demethylase and lysine hydroxylase |
| JMJD7 | ENSG00000243789 | jumonji domain containing 7 |
| JMJD8 | ENSG00000161999 | jumonji domain containing 8 |
| KAT2A | ENSG00000108773 | lysine acetyltransferase 2A |
| KAT2B | ENSG00000114166 | lysine acetyltransferase 2B |
| KAT5 | ENSG00000172977 | lysine acetyltransferase 5 |
| KAT6A | ENSG00000083168 | lysine acetyltransferase 6A |
| KAT6B | ENSG00000156650 | lysine acetyltransferase 6B |
| KAT7 | ENSG00000136504 | lysine acetyltransferase 7 |
| KAT8 | ENSG00000103510 | lysine acetyltransferase 8 |
| KDM1A | ENSG00000004487 | lysine demethylase 1A |
| KDM1B | ENSG00000165097 | lysine demethylase 1B |
| KDM2A | ENSG00000173120 | lysine demethylase 2A |
| KDM2B | ENSG00000089094 | lysine demethylase 2B |
| KDM3A | ENSG00000115548 | lysine demethylase 3A |
| KDM3B | ENSG00000120733 | lysine demethylase 3B |
| KDM4A | ENSG00000066135 | lysine demethylase 4A |
| KDM4B | ENSG00000127663 | lysine demethylase 4B |
| KDM4C | ENSG00000107077 | lysine demethylase 4C |
| KDM4D | ENSG00000186280 | lysine demethylase 4D |
| KDM4E | ENSG00000235268 | lysine demethylase 4E |
| KDM5A | ENSG00000073614 | lysine demethylase 5A |
| KDM5B | ENSG00000117139 | lysine demethylase 5B |
| KDM5C | ENSG00000126012 | lysine demethylase 5C |
| KDM5D | ENSG00000012817 | lysine demethylase 5D |
| KDM6A | ENSG00000147050 | lysine demethylase 6A |
| KDM6B | ENSG00000132510 | lysine demethylase 6B |
| KDM8 | ENSG00000155666 | lysine demethylase 8 |
| KMT2A | ENSG00000118058 | lysine methyltransferase 2A |
| KMT2B | ENSG00000272333 | lysine methyltransferase 2B |
| KMT2C | ENSG00000055609 | lysine methyltransferase 2C |
| KMT2D | ENSG00000167548 | lysine methyltransferase 2D |
| KMT2E | ENSG00000005483 | lysine methyltransferase 2E (inactive) |
| L3MBTL1 | ENSG00000185513 | L3MBTL histone methyl-lysine binding protein 1 |
| L3MBTL2 | ENSG00000100395 | L3MBTL histone methyl-lysine binding protein 2 |
| L3MBTL3 | ENSG00000198945 | L3MBTL histone methyl-lysine binding protein 3 |
| L3MBTL4 | ENSG00000154655 | L3MBTL histone methyl-lysine binding protein 4 |
| LEO1 | ENSG00000166477 | LEO1 homolog, Paf1/RNA polymerase II complex component |
| LRWD1 | ENSG00000161036 | leucine rich repeats and WD repeat domain containing 1 |
| MBD1 | ENSG00000141644 | methyl-CpG binding domain protein 1 |
| MBD2 | ENSG00000134046 | methyl-CpG binding domain protein 2 |
| MBD3 | ENSG00000071655 | methyl-CpG binding domain protein 3 |
| MBD4 | ENSG00000129071 | methyl-CpG binding domain 4, DNA glycosylase |
| MBD5 | ENSG00000204406 | methyl-CpG binding domain protein 5 |
| MBD6 | ENSG00000166987 | methyl-CpG binding domain protein 6 |
| MBTD1 | ENSG00000011258 | mbt domain containing 1 |
| MECP2 | ENSG00000169057 | methyl-CpG binding protein 2 |
| MEN1 | ENSG00000133895 | menin 1 |
| MGMT | ENSG00000170430 | O-6-methylguanine-DNA methyltransferase |
| RIOX2 | ENSG00000170854 | Ribosomal Oxygenase 2 |
| MLLT10 | ENSG00000078403 | MLLT10 histone lysine methyltransferase DOT1L cofactor |
| MLLT6 | ENSG00000275023 | MLLT6, PHD finger containing |
| MORC3 | ENSG00000159256 | MORC family CW-type zinc finger 3 |
| MORC4 | ENSG00000133131 | MORC family CW-type zinc finger 4 |
| MORF4L1 | ENSG00000185787 | mortality factor 4 like 1 |
| MORF4L2 | ENSG00000123562 | mortality factor 4 like 2 |
| MPHOSPH8 | ENSG00000196199 | M-phase phosphoprotein 8 |
| MSL3 | ENSG00000005302 | MSL Complex Subunit 3 |
| MTA1 | ENSG00000182979 | metastasis associated 1 |
| MTA2 | ENSG00000149480 | metastasis associated 1 family member 2 |
| MTF2 | ENSG00000143033 | metal response element binding transcription factor 2 |
| MYSM1 | ENSG00000162601 | Myb like, SWIRM and MPN domains 1 |
| NAP1L1 | ENSG00000187109 | nucleosome assembly protein 1 like 1 |
| NAP1L2 | ENSG00000186462 | nucleosome assembly protein 1 like 2 |
| NAP1L3 | ENSG00000186310 | nucleosome assembly protein 1 like 3 |
| NAP1L4 | ENSG00000205531 | nucleosome assembly protein 1 like 4 |
| NAP1L5 | ENSG00000177432 | nucleosome assembly protein 1 like 5 |
| NAP1L6 | ENSG00000204118 | Nucleosome Assembly Protein 1 Like 6 |
| NAT10 | ENSG00000135372 | N-acetyltransferase 10 |
| NCOA1 | ENSG00000084676 | nuclear receptor coactivator 1 |
| NCOA2 | ENSG00000140396 | nuclear receptor coactivator 2 |
| NCOA3 | ENSG00000124151 | nuclear receptor coactivator 3 |
| NSD1 | ENSG00000165671 | nuclear receptor binding SET domain protein 1 |
| OGT | ENSG00000147162 | O-linked N-acetylglucosamine (GlcNAc) transferase |
| PADI2 | ENSG00000117115 | peptidyl arginine deiminase 2 |
| PADI4 | ENSG00000159339 | peptidyl arginine deiminase 4 |
| PAF1 | ENSG00000006712 | PAF1 homolog, Paf1/RNA polymerase II complex component |
| PAGR1 | ENSG00000280789 | PAXIP1 associated glutamate rich protein 1 |
| PAXIP1 | ENSG00000157212 | PAX interacting protein 1 |
| PBRM1 | ENSG00000163939 | polybromo 1 |
| PCGF1 | ENSG00000115289 | polycomb group ring finger 1 |
| PCGF2 | ENSG00000277258 | polycomb group ring finger 2 |
| PCGF3 | ENSG00000185619 | polycomb group ring finger 3 |
| PCGF5 | ENSG00000180628 | polycomb group ring finger 5 |
| PCGF6 | ENSG00000156374 | polycomb group ring finger 6 |
| PHC1 | ENSG00000111752 | polyhomeotic homolog 1 |
| PHC2 | ENSG00000134686 | polyhomeotic homolog 2 |
| PHC3 | ENSG00000173889 | polyhomeotic homolog 3 |
| PHF1 | ENSG00000112511 | PHD finger protein 1 |
| PHF10 | ENSG00000130024 | PHD finger protein 10 |
| PHF11 | ENSG00000136147 | PHD finger protein 11 |
| PHF12 | ENSG00000109118 | PHD finger protein 12 |
| PHF13 | ENSG00000116273 | PHD finger protein 13 |
| PHF14 | ENSG00000106443 | PHD finger protein 14 |
| PHF15 | ENSG00000043143 | PHD finger protein 15 |
| PHF16 | ENSG00000102221 | PHD finger protein 16 |
| PHF17 | ENSG00000077684 | PHD finger protein 17 |
| PHF19 | ENSG00000119403 | PHD finger protein 19 |
| PHF2 | ENSG00000197724 | PHD finger protein 2 |
| PHF20 | ENSG00000025293 | PHD finger protein 20 |
| PHF20L1 | ENSG00000129292 | PHD finger protein 20 like 1 |
| PHF21A | ENSG00000135365 | PHD finger protein 21A |
| PHF21B | ENSG00000056487 | PHD finger protein 21B |
| PHF23 | ENSG00000040633 | PHD finger protein 23 |
| PHF3 | ENSG00000118482 | PHD finger protein 3 |
| PHF5A | ENSG00000100410 | PHD finger protein 5A |
| PHF6 | ENSG00000156531 | PHD finger protein 6 |
| PHF7 | ENSG00000010318 | PHD finger protein 7 |
| PHF8 | ENSG00000172943 | PHD finger protein 8 |
| PHIP | ENSG00000146247 | pleckstrin homology domain interacting protein |
| PHRF1 | ENSG00000070047 | PHD and ring finger domains 1 |
| PRDM1 | ENSG00000057657 | PR/SET domain 1 |
| PRDM10 | ENSG00000170325 | PR/SET domain 10 |
| PRDM11 | ENSG00000019485 | PR/SET domain 11 |
| PRDM12 | ENSG00000130711 | PR/SET domain 12 |
| PRDM13 | ENSG00000112238 | PR/SET domain 13 |
| PRDM14 | ENSG00000147596 | PR/SET domain 14 |
| PRDM15 | ENSG00000141956 | PR/SET domain 15 |
| PRDM16 | ENSG00000142611 | PR/SET domain 16 |
| PRDM2 | ENSG00000116731 | PR/SET domain 2 |
| PRDM4 | ENSG00000110851 | PR/SET domain 4 |
| PRDM5 | ENSG00000138738 | PR/SET domain 5 |
| PRDM6 | ENSG00000061455 | PR/SET domain 6 |
| PRDM7 | ENSG00000126856 | PR/SET domain 7 |
| PRDM8 | ENSG00000152784 | PR/SET domain 8 |
| PRDM9 | ENSG00000164256 | PR/SET domain 9 |
| PRMT1 | ENSG00000126457 | protein arginine methyltransferase 1 |
| PRMT10 | ENSG00000164169 | protein arginine methyltransferase 10 |
| PRMT2 | ENSG00000160310 | protein arginine methyltransferase 2 |
| PRMT3 | ENSG00000185238 | protein arginine methyltransferase 3 |
| PRMT5 | ENSG00000100462 | protein arginine methyltransferase 5 |
| PRMT6 | ENSG00000198890 | protein arginine methyltransferase 6 |
| PRMT7 | ENSG00000132600 | protein arginine methyltransferase 7 |
| PRMT8 | ENSG00000111218 | protein arginine methyltransferase 8 |
| PROM1 | ENSG00000007062 | prominin 1 |
| PYGO1 | ENSG00000171016 | pygopus family PHD finger 1 |
| PYGO2 | ENSG00000163348 | pygopus family PHD finger 2 |
| RAG2 | ENSG00000175097 | recombination activating 2 |
| RBBP4 | ENSG00000162521 | RB binding protein 4, chromatin remodeling factor |
| RBBP5 | ENSG00000117222 | RB binding protein 5, histone lysine methyltransferase complex subunit |
| RBBP7 | ENSG00000102054 | RB binding protein 7, chromatin remodeling factor |
| RING1 | ENSG00000204227 | ring finger protein 1 |
| RNF168 | ENSG00000163961 | ring finger protein 168 |
| RNF2 | ENSG00000121481 | ring finger protein 2 |
| RNF20 | ENSG00000155827 | ring finger protein 20 |
| RNF40 | ENSG00000103549 | ring finger protein 40 |
| RNF8 | ENSG00000112130 | ring finger protein 8 |
| RSF1 | ENSG00000048649 | remodeling and spacing factor 1 |
| RUVBL1 | ENSG00000175792 | RuvB like AAA ATPase 1 |
| RUVBL2 | ENSG00000183207 | RuvB like AAA ATPase 2 |
| RYBP | ENSG00000163602 | RING1 and YY1 binding protein |
| SCMH1 | ENSG00000010803 | Scm polycomb group protein homolog 1 |
| SCML2 | ENSG00000102098 | Scm polycomb group protein like 2 |
| SET | ENSG00000119335 | SET nuclear proto-oncogene |
| SETD1A | ENSG00000099381 | SET domain containing 1A, histone lysine methyltransferase |
| SETD1B | ENSG00000139718 | SET domain containing 1B, histone lysine methyltransferase |
| SETD2 | ENSG00000181555 | SET domain containing 2, histone lysine methyltransferase |
| SETD3 | ENSG00000183576 | SET domain containing 3, actin histidine methyltransferase |
| SETD4 | ENSG00000185917 | SET domain containing 4 |
| SETD5 | ENSG00000168137 | SET domain containing 5 |
| SETD6 | ENSG00000103037 | SET domain containing 6, protein lysine methyltransferase |
| SETD7 | ENSG00000145391 | SET domain containing 7, histone lysine methyltransferase |
| SETD8 | ENSG00000183955 | SET Domain-Containing Protein 8 |
| SETD9 | ENSG00000155542 | SET domain containing 9 |
| SETDB1 | ENSG00000143379 | SET domain bifurcated histone lysine methyltransferase 1 |
| SETDB2 | ENSG00000136169 | SET domain bifurcated histone lysine methyltransferase 2 |
| SETMAR | ENSG00000170364 | SET domain and mariner transposase fusion gene |
| SFMBT1 | ENSG00000163935 | Scm like with four mbt domains 1 |
| SFMBT2 | ENSG00000198879 | Scm like with four mbt domains 2 |
| SHPRH | ENSG00000146414 | SNF2 histone linker PHD RING helicase |
| SIN3A | ENSG00000169375 | SIN3 transcription regulator family member A |
| SIN3B | ENSG00000127511 | SIN3 transcription regulator family member B |
| SIRT1 | ENSG00000096717 | sirtuin 1 |
| SIRT2 | ENSG00000068903 | sirtuin 2 |
| SIRT6 | ENSG00000077463 | sirtuin 6 |
| SIRT7 | ENSG00000187531 | sirtuin 7 |
| SMARCA1 | ENSG00000102038 | SWI/SNF related, matrix associated, actin dependent regulator of chromatin, subfamily a, member 1 |
| SMARCA2 | ENSG00000080503 | SWI/SNF related, matrix associated, actin dependent regulator of chromatin, subfamily a, member 2 |
| SMARCA4 | ENSG00000127616 | SWI/SNF related, matrix associated, actin dependent regulator of chromatin, subfamily a, member 4 |
| SMARCA5 | ENSG00000153147 | SWI/SNF related, matrix associated, actin dependent regulator of chromatin, subfamily a, member 5 |
| SMARCAD1 | ENSG00000163104 | SWI/SNF-related, matrix-associated actin-dependent regulator of chromatin, subfamily a, containing DEAD/H box 1 |
| SMARCAL1 | ENSG00000138375 | SWI/SNF related, matrix associated, actin dependent regulator of chromatin, subfamily a like 1 |
| SMARCB1 | ENSG00000099956 | SWI/SNF related, matrix associated, actin dependent regulator of chromatin, subfamily b, member 1 |
| SMARCC1 | ENSG00000173473 | SWI/SNF related, matrix associated, actin dependent regulator of chromatin subfamily c member 1 |
| SMARCC2 | ENSG00000139613 | SWI/SNF related, matrix associated, actin dependent regulator of chromatin subfamily c member 2 |
| SMARCD1 | ENSG00000066117 | SWI/SNF related, matrix associated, actin dependent regulator of chromatin, subfamily d, member 1 |
| SMARCD2 | ENSG00000108604 | SWI/SNF related, matrix associated, actin dependent regulator of chromatin, subfamily d, member 2 |
| SMARCD3 | ENSG00000082014 | SWI/SNF related, matrix associated, actin dependent regulator of chromatin, subfamily d, member 3 |
| SMARCE1 | ENSG00000073584 | SWI/SNF related, matrix associated, actin dependent regulator of chromatin, subfamily e, member 1 |
| SMYD1 | ENSG00000115593 | SET and MYND domain containing 1 |
| SMYD2 | ENSG00000143499 | SET and MYND domain containing 2 |
| SMYD3 | ENSG00000185420 | SET and MYND domain containing 3 |
| SMYD4 | ENSG00000186532 | SET and MYND domain containing 4 |
| SMYD5 | ENSG00000135632 | SMYD family member 5 |
| SP100 | ENSG00000067066 | SP100 nuclear antigen |
| SP110 | ENSG00000135899 | SP110 nuclear body protein |
| SP140 | ENSG00000079263 | SP140 nuclear body protein |
| SP140L | ENSG00000185404 | SP140 nuclear body protein like |
| SRCAP | ENSG00000080603 | Snf2 related CREBBP activator protein |
| SSRP1 | ENSG00000149136 | structure specific recognition protein 1 |
| SUPT16H | ENSG00000092201 | SPT16 homolog, facilitates chromatin remodeling subunit |
| SUPT6H | ENSG00000109111 | SPT6 homolog, histone chaperone and transcription elongation factor |
| SUPT7L | ENSG00000119760 | SPT7 like, STAGA complex subunit gamma |
| SUV39H1 | ENSG00000101945 | suppressor of variegation 3-9 homolog 1 |
| SUV39H2 | ENSG00000152455 | suppressor of variegation 3-9 homolog 2 |
| SUV420H1 | ENSG00000110066 | Suppressor Of Variegation 4-20 Homolog 1 |
| SUV420H2 | ENSG00000133247 | Suppressor Of Variegation 4-20 Homolog 2 |
| SUZ12 | ENSG00000178691 | SUZ12 polycomb repressive complex 2 subunit |
| TADA3L | ENSG00000171148 | Transcriptional Adapter 3-Like |
| TAF1 | ENSG00000147133 | TATA-box binding protein associated factor 1 |
| TAF1L | ENSG00000122728 | TATA-box binding protein associated factor 1 like |
| TAF3 | ENSG00000165632 | TATA-box binding protein associated factor 3 |
| TAF8 | ENSG00000137413 | TATA-box binding protein associated factor 8 |
| TDRD3 | ENSG00000083544 | tudor domain containing 3 |
| TET1 | ENSG00000138336 | tet methylcytosine dioxygenase 1 |
| TET2 | ENSG00000168769 | tet methylcytosine dioxygenase 2 |
| TET3 | ENSG00000187605 | tet methylcytosine dioxygenase 3 |
| TRIM24 | ENSG00000122779 | tripartite motif containing 24 |
| TRIM28 | ENSG00000130726 | tripartite motif containing 28 |
| TRIM33 | ENSG00000197323 | tripartite motif containing 33 |
| TRIM66 | ENSG00000166436 | tripartite motif containing 66 |
| TRRAP | ENSG00000196367 | transformation/transcription domain associated protein |
| UHRF1 | ENSG00000276043 | ubiquitin like with PHD and ring finger domains 1 |
| UHRF2 | ENSG00000147854 | ubiquitin like with PHD and ring finger domains 2 |
| USP22 | ENSG00000124422 | ubiquitin specific peptidase 22 |
| UTY | ENSG00000183878 | ubiquitously transcribed tetratricopeptide repeat containing, Y-linked |
| WDR5 | ENSG00000196363 | WD repeat domain 5 |
| WDR82 | ENSG00000164091 | WD repeat domain 82 |
| WHSC1 | ENSG00000109685 | Wolf-Hirschhorn Syndrome Candidate 1 |
| WHSC1L1 | ENSG00000147548 | Wolf-Hirschhorn Syndrome Candidate 1-Like 1 |
| ZCWPW1 | ENSG00000078487 | zinc finger CW-type and PWWP domain containing 1 |
| ZCWPW2 | ENSG00000206559 | zinc finger CW-type and PWWP domain containing 2 |
| ZMYND8 | ENSG00000101040 | zinc finger MYND-type containing 8 |
| ZMYND11 | ENSG00000015171 | Zinc Finger MYND-Type Containing 11 |

**Table S2 siRNAs for transient gene silencing**

| **Gene** | **Sequence (5’-3’)** | **Specie** | **Cat. no.** | **Supplier** |
| --- | --- | --- | --- | --- |
| NT5  (Non-targeting control #5) | UGGUUUACAUGUCGACUAA | - | D-001206-13 | Dharmacon |
| *Ezh2* | GGAAAGAACUGAAACCUUA  CAGAAGAGCUGAUGAAGUA  AGAAAGAUCUAGAGGAUAA  GGAGGGAGCUAAGGAGUUU | Ms | M-040882-00 | Dharmacon |
| *EZH2* | CAAAGAAUCUAGCAUCAUA  GAGGACGGCUUCCCAAUAA  GCUGAAGCCUCAAUGUUUA  GAAUGGAAACAGCGAAGGA | Hu | M-004218-03 | Dharmacon |
| *Nfatc1* | GCCAUAACUUUCUGCAAGA  GGGCAAGCAUCACGGAGGA  CCAACUACUCCUACCCAUA  ACGGUUACUUGGAGAAUGA | Ms | M-054700-01 | Dharmacon |
| *NFATc1* | UCAGAAACUCCGACAUUGA  GGACAGCUAUCCGGUCGUG  GUUGAGAUCCCGCCAUUUC  AGGAAGAACACACGGGUAC | Hu | D-003605-07  D-003605-09  D-003605-10  D-003605-23 | Dharmacon |

**Table S2: qRT-PCR primers**

| **Gene** | **Specie** | **Application** | **Primer** | **Primer sequence** |
| --- | --- | --- | --- | --- |
| ***Cdh1*** | Mouse | qRT-PCR | Fwd | CCTGGCACTGGTATCTCTTCA |
|  |  |  | Rev | AGCCATTGCCAAGTACATCCTC |
| ***Epcam*** | Mouse | qRT-PCR | Fwd | GAGTCCGAAGAACCGACAAGG |
|  |  |  | Rev | CTGATGGTCGTAGGGGCTTTC |
| ***Krt14*** | Mouse | qRT-PCR | Fwd | CAGAGCGGCAAGAGTGAGAT |
|  |  |  | Rev | TGCCGATCATCTCCTGGATCT |
| ***Krt18*** | Mouse | qRT-PCR | Fwd | AGTCTGTGGAGAGCGACATC |
|  |  |  | Rev | TCAATCCAGAGCTGGCAATC |
| ***N-cadherin*** | Mouse | qRT-PCR | Fwd | GCGCAGTCTTACCGAAGGATG |
|  |  |  | Rev | ATACACCGTGCCGTCCTCGT |
| ***Nfatc1*** | Mouse | qRT-PCR | Fwd | GCCTTTTGCGAGCAGTATCT |
|  |  |  | Rev | GCTGCCTTCCGTCTCATAGT |
| ***Rplp0*** | Mouse | qRT-PCR | Fwd | GATTCGGGATATGCTGTTGG |
|  |  |  | Rev | GCCTGGAAGAAGGAGGTCTT |
| ***Snai1*** | Mouse | qRT-PCR | Fwd | CTGGTGAGAAGCCATTCTCCT |
|  |  |  | Rev | CCTGGCACTGGTATCTCTTCA |
| ***Snai2*** | Mouse | qRT-PCR | Fwd | AACATTTCAACGCCTCCAAG |
|  |  |  | Rev | CGAGGTGAGGATCTCTGGTT |
| ***Twist1*** | Mouse | qRT-PCR | Fwd | CGGACAAGCTGAGCAAGATTC |
|  |  |  | Rev | TCCAGACGGAGAAGGCGTAG |
| ***Twist2*** | Mouse | qRT-PCR | Fwd | GGCCGCCAGGTACATAGAC |
|  |  |  | Rev | GTAGCTGAGACGCTCGTGA |
| ***Vimentin*** | Mouse | qRT-PCR | Fwd | CGGCTGCGAGAGAAATTGC |
|  |  |  | Rev | CCACTTTCCGTTCAAGGTCAAG |
| ***Zeb1*** | Mouse | qRT-PCR | Fwd | CACCAGAAGCCAGCAGTCAT |
|  |  |  | Rev | CGTTCTTCTCATGGCGGTACT |
| ***Sox2*** | Mouse | qRT-PCR | Fwd | AACGCCTTCATGGTATGGTC |
|  |  |  | Rev | CGGTCTCGGACAAAAGTTTC |
| ***Nanog*** | Mouse | qRT-PCR | Fwd | CCTGATTCTTCTACCAGTCCCA |
|  |  |  | Rev | GGCCTGAGAGAACACAGTCC |
| ***Ezh2*** | Mouse | qRT-PCR | Fwd | TCCATGCAACACCCAACACA |
|  |  |  | Rev | AACTCCTTAGCTCCCTCCAGAT |
| ***Suz12*** | Mouse | qRT-PCR | Fwd | AGCATCAAAAGCTTGTCTGCAC |
|  |  |  | Rev | ACTTTCACAAGCAGGACTTCCA |
| ***Rbbp7*** | Mouse | qRT-PCR | Fwd | ACCTGGTTATGACCCATGCT |
|  |  |  | Rev | AACTCGAGCAACCACCAGAT |
| ***Mtf2*** | Mouse | qRT-PCR | Fwd | TTCAGAAGCTCCCAATGAAA |
|  |  |  | Rev | TTGTTGCAAAAACGCACTGT |

**Table S3: ChIP-qPCR primers**

| **Gene** | **Specie** | **Application** | **Primer** | **Primer sequence** |
| --- | --- | --- | --- | --- |
| ***Hmga2_ChIP_TSS_H3K27ac*** | Mouse | ChIP-qPCR | Fwd | CGTACTGACTTGCTGCTGCT |
|  |  |  | Rev | ACCGGTAGAGGCAGTGGTAG |
| ***Hmga2_ChIP_TSS_H3K27me3*** | Mouse | ChIP-qPCR | Fwd | CTGCAGGAGAAGCAAGAGGA |
|  |  |  | Rev | CCTTCGAGGCAGTTGTGTTT |
| ***Nfatc1_ChIP_TSS_H3K27ac*** | Mouse | ChIP-qPCR | Fwd | CAGCATCTAGCGGCTCTTTC |
|  |  |  | Rev | CCTGAGACATGTTTGGCACT |
| ***Nfatc1_ChIP_TSS_H3K27me3*** | Mouse | ChIP-qPCR | Fwd | AAATTCCCATCCCGCTAAAT |
|  |  |  | Rev | CTCGTACAGCAAGCAATCCA |

**Table S4: Primary antibodies used in this study**

| **Antibody** | **Cat. no./Clone. no.** | **Source** | **Western blot** | **IHC** | **IF** | **ChIP** | **FACS** |
| --- | --- | --- | --- | --- | --- | --- | --- |
| Actin B | 8224 | Abcam | 1:2 000 |  |  |  |  |
| E-cadherin | 24E10 | Cell Signaling | 1:1 000 |  |  |  |  |
| EZH2 | 5246/ D2C9 | Cell Signalling | 1:1 000 | 1:150 | 1:100 |  |  |
| FITC-α-EpCAM | 118207 | Biolegend |  |  |  |  | 1:400 |
| H3 | 601902 | Biolegend | 1:500 |  |  |  |  |
| H3K27ac | C15410196 | Diagenode |  |  |  | 2 μg |  |
| H3K27me3 | C15410195 | Diagenode |  |  |  | 2 μg |  |
| N-cadherin | D4R1H | Cell Signaling | 1:1 000 |  |  |  |  |
| NFATc1 | 649601 | Biolegend | 1:500 | 1:25 |  |  |  |
| NFATc1 | DF6446 | Affinity Biosciences |  | 1:50 |  |  |  |
| SUZ12 | D39F6/ 3737 | Cell Signalling | 1:1 000 |  | 1:100 |  |  |
| Vimentin | 18814 | Cell Signaling | 1:1 000 |  |  |  |  |

**Table S5: Secondary antibodies used in this study**

| **Antibody** | **Conjugate** | **Cat. No.** | **Source** | **Western blot** | **IHC** | **IF** |
| --- | --- | --- | --- | --- | --- | --- |
| Anti-mouse IgG | HRP | 211-032-171 | Dianova | 1:10 000 |  |  |
| Anti-mouse IgG | Biotin | 711-065-150 | Dianova |  | 1:200 |  |
| Anti-rabbit IgG | HRP | 211-032-171 | Dianova | 1:10 000 |  |  |
| Anti-rabbit IgG | Biotin | 711-065-152 | Dianova |  | 1:200 |  |
| Anti-rabbit IgG | Alexa 555 | A-31570 | Molecular Probes |  |  | 1:400 |
