# Supplementary figures and images for: Decreased PRC2 activity supports the survival of basal-like breast cancer cells to cytotoxic treatments
Source: Cell Death Dis. 2021 Nov 29;12(12):1118. doi: 10.1038/s41419-021-04407-y (PMC8630036; doi:10.1038/s41419-021-04407-y)

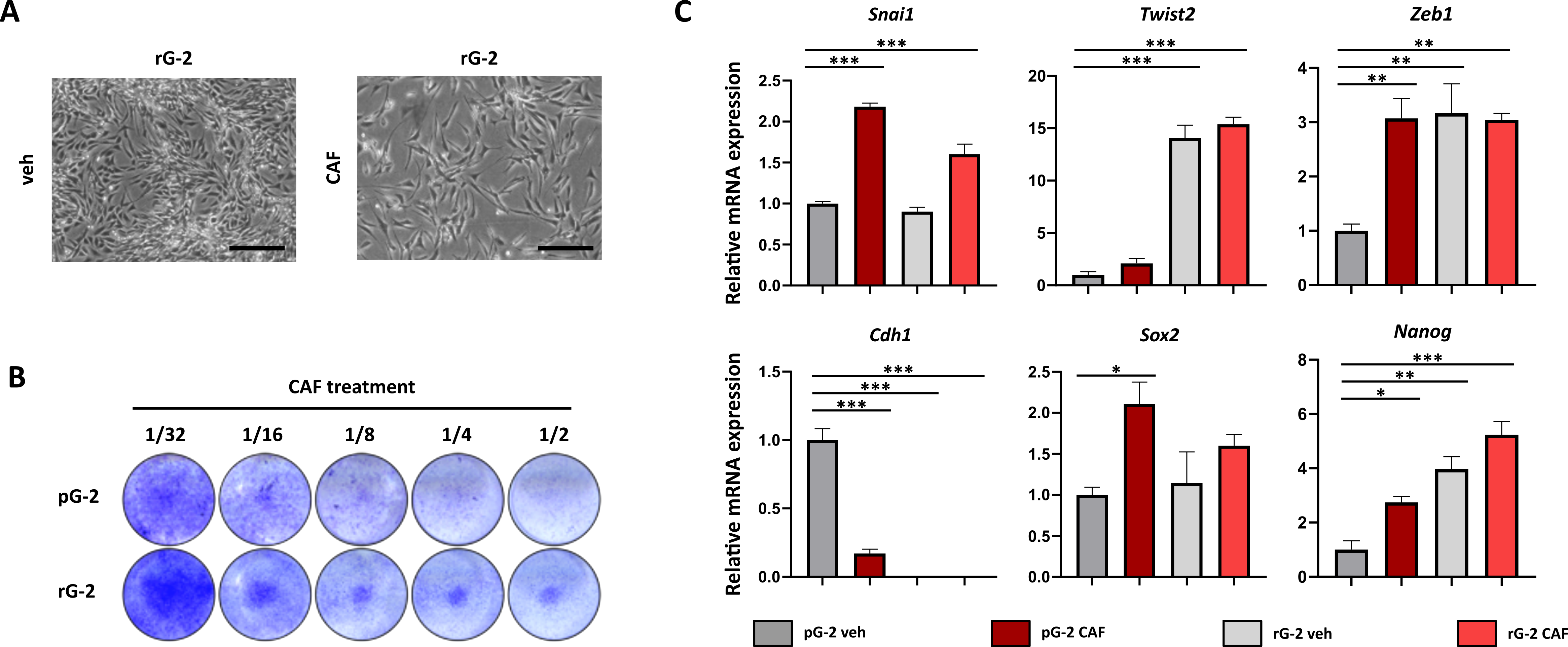

Supplement: Supplementary file 4 — Figure S1 [file 41419_2021_4407_MOESM4_ESM.tif]

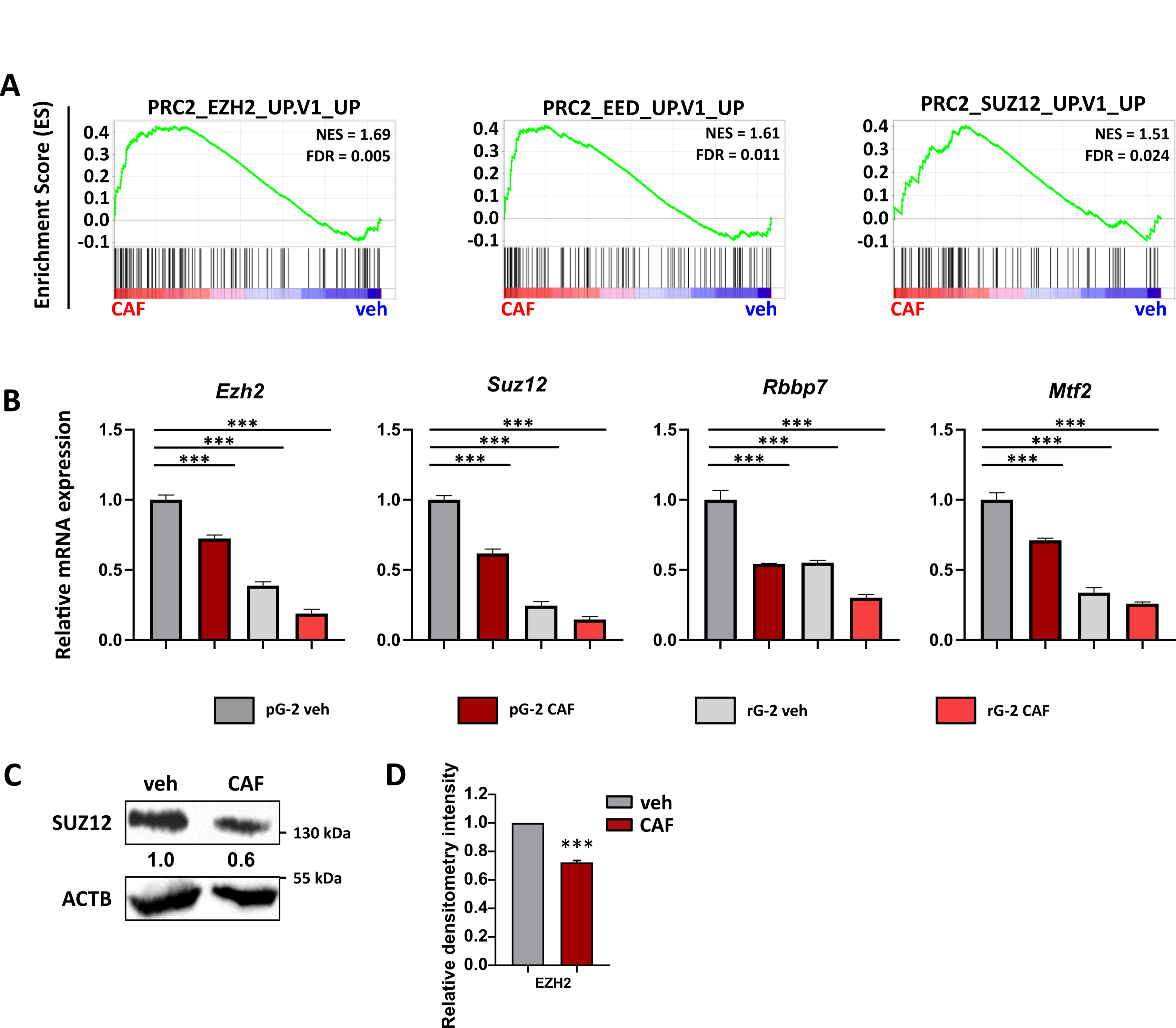

Supplement: Supplementary file 5 — Figure S2 [file 41419_2021_4407_MOESM5_ESM.tif]

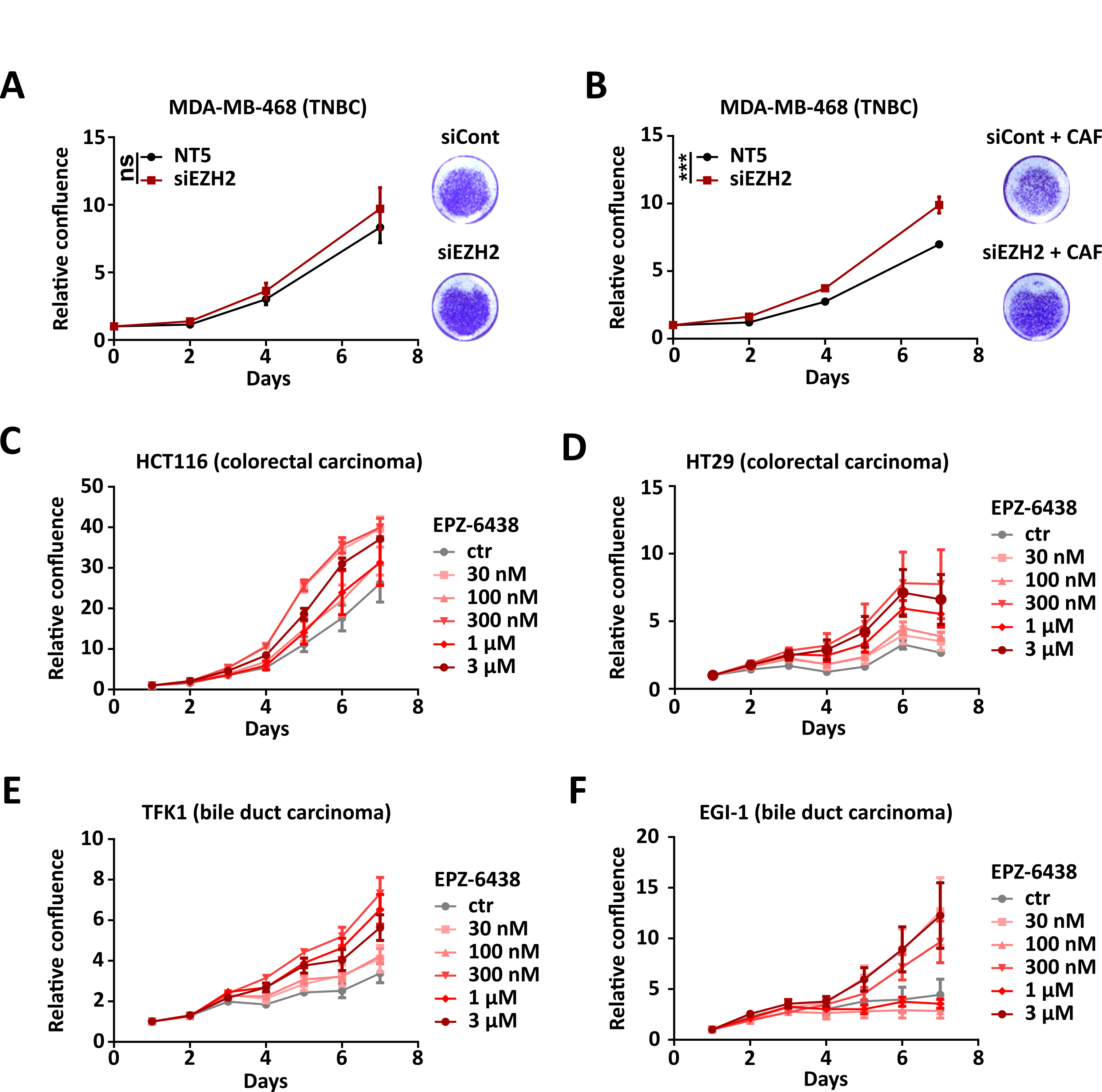

Supplement: Supplementary file 6 — Figure S3 [file 41419_2021_4407_MOESM6_ESM.tif]

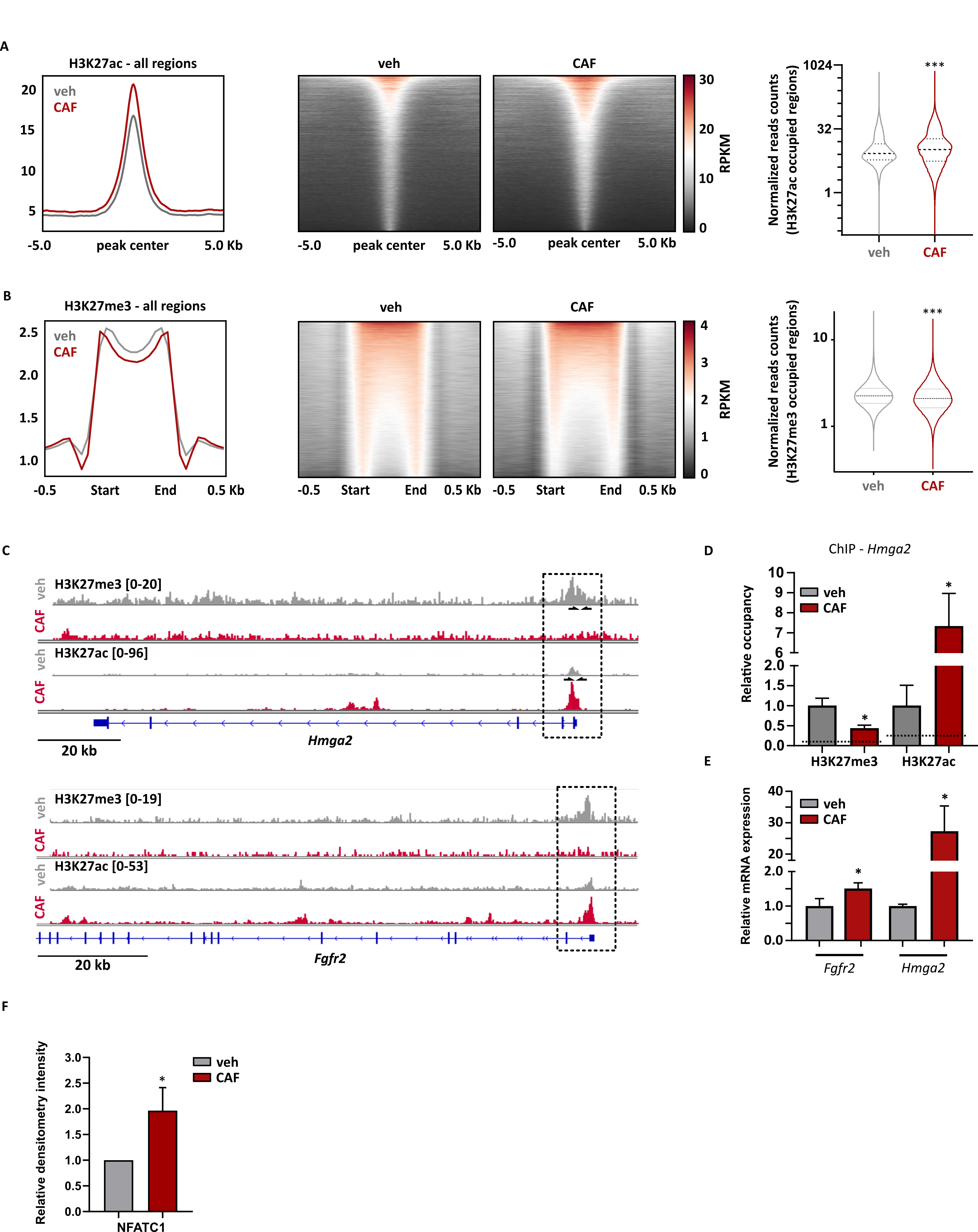

Supplement: Supplementary file 7 — Figure S4 [file 41419_2021_4407_MOESM7_ESM.tif]

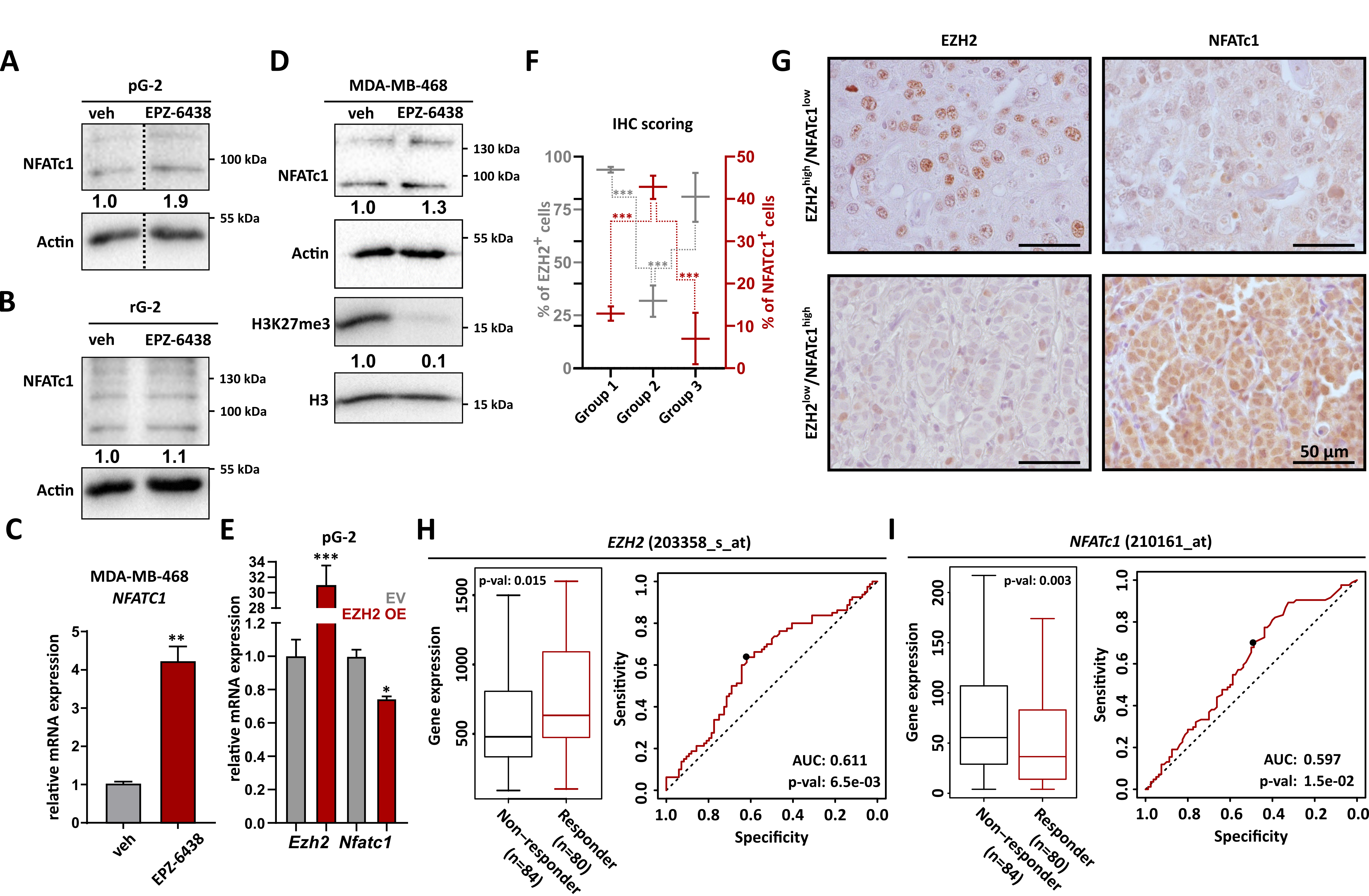

Supplement: Supplementary file 8 — Figure S5 [file 41419_2021_4407_MOESM8_ESM.tif]

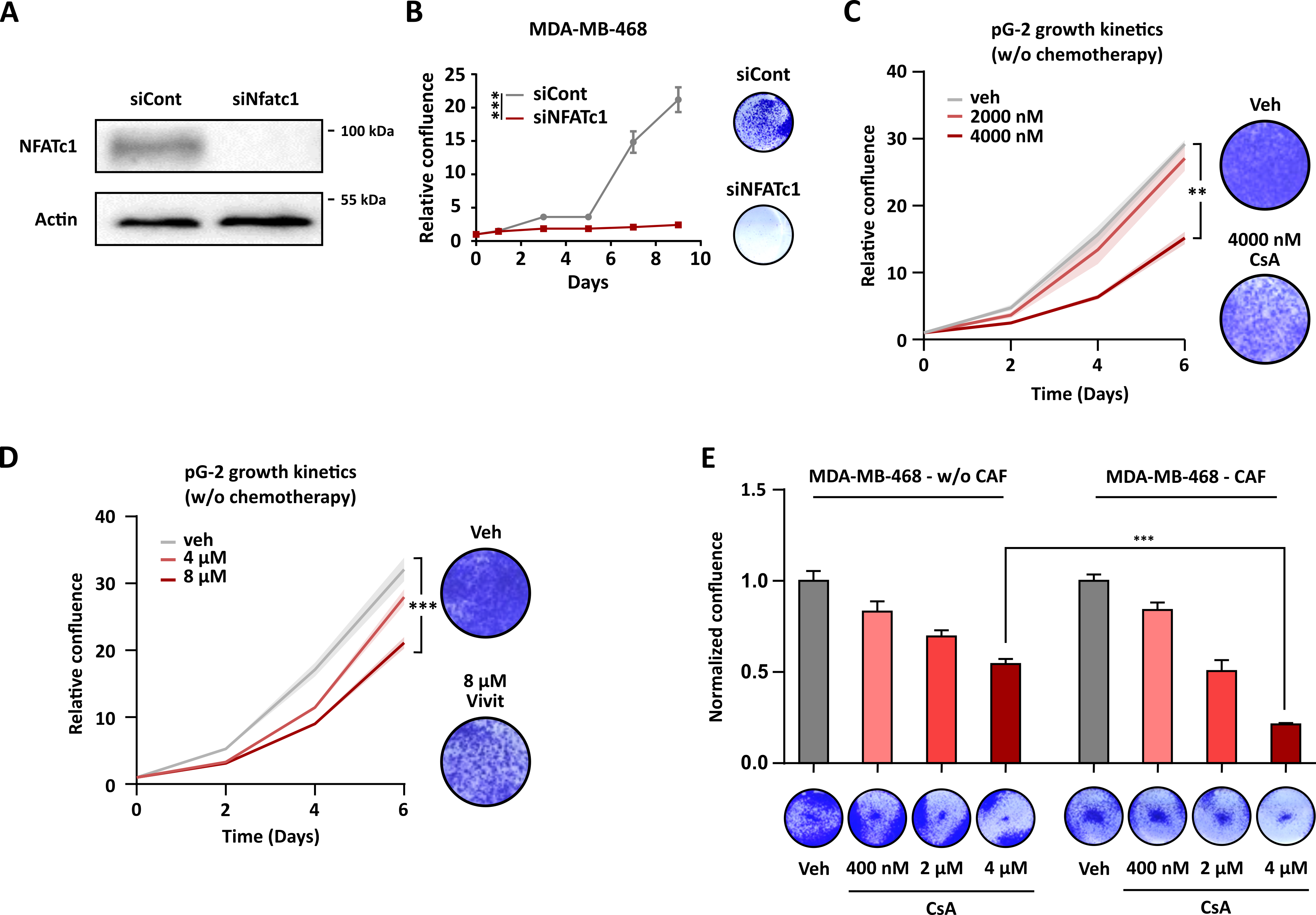

Supplement: Supplementary file 9 — Figure S6 [file 41419_2021_4407_MOESM9_ESM.tif]
