# Supplementary material for: Decreased PRC2 activity supports the survival of basal-like breast cancer cells to cytotoxic treatments
Source: Cell Death Dis. 2021 Nov 29;12(12):1118. doi: 10.1038/s41419-021-04407-y (PMC8630036; doi:10.1038/s41419-021-04407-y)
Supplement: Supplementary file 1 — Authorship agreement [file 41419_2021_4407_MOESM1_ESM.pdf]

Von: Iga M <iga.mieczkowska92@gmail.com>
Gesendet: Freitag, 5. November 2021 10:05
An: Wegwitz, Florian
Betreff: Re: Mieczkowska et al. - CDDIS-21-0618RR Initial Quality Check

Dear Florian,
I accept it.

Dr. Iga Mieczkowska

czw., 4 lis 2021 o 14:15 Wegwitz, Florian <fwegwit@gwdg.de> napisał(a):

> Dear co-authors,
>
> I just received an Email from the *Cell Death & Disease* editorial office asking me kindly to provide from all authors a statement that they agree with the accepted version of the manuscript. Therefore, I would be very happy if you could send me a short email with your confirmation so that we can proceed with the further step.
> Thank you all very much.
>
> Best wishes,
>
> Florian
>
> --

> Dr. Florian Wegwitz
> Group Leader
> Laboratory for Molecular Gynecology
> University Medical Center Göttingen
> Department of Gynecology and Obstetrics
> Georg-August-University
> Robert-Koch-Straße 40
> 37075 Göttingen
> Email: fwegwit@gwdg.de, florian.wegwitz@med.uni-goettingen.de
> Tel.: +49-(0)551-39 69811

Von: Pantelaiou Prokaki, Garyfallia
Gesendet: Donnerstag, 4. November 2021 16:42
An: Wegwitz, Florian
Betreff: Απ: Mieczkowska et al. - CDDIS-21-0618RR Initial Quality Check

Dear Dr. Florian Wegwitz,

I would like to inform you that I fully agree with the accepted version of the manuscript "Decreased PRC2 activity supports the survival of basal-like breast cancer cells to cytotoxic treatments." by Iga Mieczkowska, Garyfallia Pantelaiou-Prokaki, Evangelos Prokakis, Geske Schmidt, Lukas Müller-Kirschbaum, Marcel Werner, Madhobi Sen, Taras Velychko, Katharina Jannasch, Christian Dullin, Joanna Napp, Klaus Pantel, Harriet Wikman, Maria Wiese, Christof Kramm, Frauke Alves, and Florian Wegwitz [Paper #CDDIS-21-0618RR], for publication in *Cell Death & Disease.*

With the best wishes,

Garyfallia Pantelaiou-Prokaki

____

Garyfallia Pantelaiou-Prokaki
Ph.D. Student

Laboratory for Molecular Gynecology
University Medical Center Göttingen
Department of Gynecology and Obstetrics
Georg-August-University
Robert-Koch-Straße 40
37075 Göttingen

&

Translational Molecular Imaging
Max Planck Institute for Experimental Medicine
Hermann-Rein-Straße 3
37075 Göttingen

Από: Wegwitz, Florian
Στάλθηκε: Πέμπτη, 4 Νοεμβρίου 2021 3:28:15 μμ
Προς: Iga Mieczkowska; Pantelaiou Prokaki, Garyfallia; Prokakis, Evangelos; Schmidt, Geske Elisabeth; Müller-Kirschbaum, Lukas Christoph; Marcel.Werner@campus.lmu.de; Madhobi Sen; Taras Velychko; Jannasch, Katharina; Dullin, Christian; Napp, Joanna; pantel@uke.de; Wikman; Wiese, Maria; Kramm, Christof; Alves, Frauke
Θέμα: AW: Mieczkowska et al. - CDDIS-21-0618RR Initial Quality Check

Dear all,

Sorry, the files were missing in the previous email.
Attached the accepted manuscript files (these are the same as for the resubmission).
I wish a good afternoon!

Best regards,

Florian Wegwitz

--
Dr. Florian Wegwitz
Group Leader
Laboratory for Molecular Gynecology
University Medical Center Göttingen
Department of Gynecology and Obstetrics
Georg-August-University
Robert-Koch-Straße 40
37075 Göttingen
Email: fwegwit@gwdg.de, florian.wegwitz@med.uni-goettingen.de
Tel.: +49-(0)551-39 69811

Von: Wegwitz, Florian
Gesendet: Donnerstag, 4. November 2021 14:15
An: 'Iga Mieczkowska' <iga.mieczkowska92@gmail.com>; Pantelaiou Prokaki, Garyfallia <garyfallia.prokaki@em.mpg.de>; Prokakis, Evangelos <eprokak@gwdg.de>; Schmidt, Geske Elisabeth <geske.schmidt@med.uni-goettingen.de>; Müller-Kirschbaum, Lukas Christoph <lukas.mueller-kirschbaum@med.uni-goettingen.de>; 'Marcel.Werner@campus.lmu.de' <Marcel.Werner@campus.lmu.de>; 'Madhobi Sen' <senmadhobi@gmail.com>; 'Taras Velychko' <taras.velychko@gmail.com>; Jannasch, Katharina <katharina.jannasch@zvw.uni-goettingen.de>; Dullin, Christian <christian.dullin@med.uni-goettingen.de>; Napp, Joanna <napp@em.mpg.de>; 'pantel@uke.de' <pantel@uke.de>; 'Wikman' <h.wikman@uke.de>; Wiese, Maria <maria.wiese@med.uni-goettingen.de>; Kramm, Christof <christof.kramm@med.uni-goettingen.de>; Alves, Frauke <falves@gwdg.de>
Betreff: Mieczkowska et al. - CDDIS-21-0618RR Initial Quality Check

Dear co-authors,

I just received an Email from the *Cell Death & Disease* editorial office asking me kindly to provide from all authors a statement that they agree with the accepted version of the manuscript. Therefore, I would be very happy if you could send me a short email with your confirmation so that we can proceed with the further step.
Thank you all very much.

Best wishes,

Florian

--
Dr. Florian Wegwitz
Group Leader
Laboratory for Molecular Gynecology
University Medical Center Göttingen
Department of Gynecology and Obstetrics
Georg-August-University
Robert-Koch-Straße 40
37075 Göttingen
Email: fwegwit@gwdg.de, florian.wegwitz@med.uni-goettingen.de
Tel.: +49-(0)551-39 69811

Von: Prokakis, Evangelos
Gesendet: Donnerstag, 4. November 2021 14:47
An: Wegwitz, Florian
Betreff: RE: Mieczkowska et al. - CDDIS-21-0618RR Initial Quality Check

Dear Florian,

With the current email, I would like to inform you that I fully agree with the accepted version of the manuscript for publication at *Cell Death and Disease*.

With best wishes,

Evangelos Prokakis

Evangelos Prokakis, *Dr. rer. nat.*
Molecular Gynecology Lab
Department of Gynecology and Obstetrics
Medical School of Göttingen
Robert-Koch Str. 40
37075, Göttingen
Niedersachsen, Germany
Email: eprokak@gwdg.de

From: Wegwitz, Florian
Sent: Thursday, November 4, 2021 2:15 PM
To: Iga Mieczkowska <iga.mieczkowska92@gmail.com>; Pantelaiou Prokaki, Garyfallia <garyfallia.prokaki@em.mpg.de>; Prokakis, Evangelos <eprokak@gwdg.de>; Schmidt, Geske Elisabeth <geske.schmidt@med.uni-goettingen.de>; Müller-Kirschbaum, Lukas Christoph <lukas.mueller-kirschbaum@med.uni-goettingen.de>; Marcel.Werner@campus.lmu.de; Madhobi Sen <senmadhobi@gmail.com>; Taras Velychko <taras.velychko@gmail.com>; Jannasch, Katharina <katharina.jannasch@zvw.uni-goettingen.de>; Dullin, Christian <christian.dullin@med.uni-goettingen.de>; Napp, Joanna <napp@em.mpg.de>; pantel@uke.de; Wikman <h.wikman@uke.de>; Wiese, Maria <maria.wiese@med.uni-goettingen.de>; Kramm, Christof <christof.kramm@med.uni-goettingen.de>; Alves, Frauke <falves@gwdg.de>
Subject: Mieczkowska et al. - CDDIS-21-0618RR Initial Quality Check

Dear co-authors,

I just received an Email from the *Cell Death & Disease* editorial office asking me kindly to provide from all authors a statement that they agree with the accepted version of the manuscript. Therefore, I would be very happy if you could send me a short email with your confirmation so that we can proceed with the further step.
Thank you all very much.

Best wishes,

Florian

--
Dr. Florian Wegwitz
Group Leader
Laboratory for Molecular Gynecology
University Medical Center Göttingen
Department of Gynecology and Obstetrics
Georg-August-University
Robert-Koch-Straße 40
37075 Göttingen
Email: fwegwit@gwdg.de, florian.wegwitz@med.uni-goettingen.de
Tel.: +49-(0)551-39 69811

Von: Schmidt, Geske Elisabeth <geske.schmidt@med.uni-goettingen.de>
Gesendet: Donnerstag, 4. November 2021 16:29
An: Wegwitz, Florian
Betreff: AW: Mieczkowska et al. - CDDIS-21-0618RR Initial Quality Check

Hi Florian,

I agree with the accepted version of the manuscript.

Best wishes,
Geske

Von: Wegwitz, Florian <fwegwit@gwdg.de>
Gesendet: Donnerstag, 4. November 2021 15:28
An: Iga Mieczkowska <iga.mieczkowska92@gmail.com>; Pantelaiou Prokaki, Garyfallia <garyfallia.prokaki@em.mpg.de>; Prokakis, Evangelos <eprokak@gwdg.de>; Schmidt, Geske Elisabeth <geske.schmidt@med.uni-goettingen.de>; Müller-Kirschbaum, Lukas Christoph <lukas.mueller-kirschbaum@med.uni-goettingen.de>; Marcel.Werner@campus.lmu.de; Madhobi Sen <senmadhobi@gmail.com>; Taras Velychko <taras.velychko@gmail.com>; Jannasch, Katharina <katharina.jannasch@zvw.uni-goettingen.de>; Dullin, Christian <christian.dullin@med.uni-goettingen.de>; Napp, Joanna <napp@em.mpg.de>; pantel@uke.de; Wikman <h.wikman@uke.de>; Wiese, Maria <maria.wiese@med.uni-goettingen.de>; Kramm, Christof <christof.kramm@med.uni-goettingen.de>; Alves, Frauke <falves@gwdg.de>
Betreff: AW: Mieczkowska et al. - CDDIS-21-0618RR Initial Quality Check

Dear all,

Sorry, the files were missing in the previous email.
Attached the accepted manuscript files (these are the same as for the resubmission).
I wish a good afternoon!

Best regards,

Florian Wegwitz

--
Dr. Florian Wegwitz
Group Leader
Laboratory for Molecular Gynecology
University Medical Center Göttingen
Department of Gynecology and Obstetrics
Georg-August-University
Robert-Koch-Straße 40
37075 Göttingen
Email: fwegwit@gwdg.de, florian.wegwitz@med.uni-goettingen.de
Tel.: +49-(0)551-39 69811

Von: Wegwitz, Florian
Gesendet: Donnerstag, 4. November 2021 14:15
An: 'Iga Mieczkowska' <iga.mieczkowska92@gmail.com>; Pantelaiou Prokaki, Garyfallia <garyfallia.prokaki@em.mpg.de>; Prokakis, Evangelos <eprokak@gwdg.de>; Schmidt, Geske Elisabeth <geske.schmidt@med.uni-goettingen.de>; Müller-Kirschbaum, Lukas Christoph <lukas.mueller-kirschbaum@med.uni-goettingen.de>; 'Marcel.Werner@campus.lmu.de' <Marcel.Werner@campus.lmu.de>; 'Madhobi Sen' <senmadhobi@gmail.com>; 'Taras Velychko' <taras.velychko@gmail.com>; Jannasch, Katharina <katharina.jannasch@zvw.uni-goettingen.de>; Dullin, Christian <christian.dullin@med.uni-goettingen.de>; Napp, Joanna <napp@em.mpg.de>; 'pantel@uke.de' <pantel@uke.de>; 'Wikman' <h.wikman@uke.de>; Wiese, Maria <maria.wiese@med.uni-goettingen.de>; Kramm, Christof <christof.kramm@med.uni-goettingen.de>; Alves, Frauke <falves@gwdg.de>
Betreff: Mieczkowska et al. - CDDIS-21-0618RR Initial Quality Check

Dear co-authors,

I just received an Email from the *Cell Death & Disease* editorial office asking me kindly to provide from all authors a statement that they agree with the accepted version of the manuscript. Therefore, I would be very happy if you could send me a short email with your confirmation so that we can proceed with the further step.
Thank you all very much.

Best wishes,

Florian

--
Dr. Florian Wegwitz
Group Leader
Laboratory for Molecular Gynecology
University Medical Center Göttingen
Department of Gynecology and Obstetrics
Georg-August-University
Robert-Koch-Straße 40
37075 Göttingen
Email: fwegwit@gwdg.de, florian.wegwitz@med.uni-goettingen.de
Tel.: +49-(0)551-39 69811

Von: Müller-Kirschbaum, Lukas Christoph <lukas.mueller-kirschbaum@med.uni-goettingen.de>
Gesendet: Donnerstag, 4. November 2021 14:26
An: Wegwitz, Florian
Betreff: RE: Mieczkowska et al. - CDDIS-21-0618RR Initial Quality Check

Hey Florian,

I agree with the accepted version of the manuscript and I am very pleased for the opportunity to contribute to the study.
Good luck and best wishes,

Lukas

From: Wegwitz, Florian <fwegwit@gwdg.de>
Sent: Thursday, 4 November 2021 14:15
To: Iga Mieczkowska <iga.mieczkowska92@gmail.com>; Pantelaiou Prokaki, Garyfallia <garyfallia.prokaki@em.mpg.de>; Prokakis, Evangelos <eprokak@gwdg.de>; Schmidt, Geske Elisabeth <geske.schmidt@med.uni-goettingen.de>; Müller-Kirschbaum, Lukas Christoph <lukas.mueller-kirschbaum@med.uni-goettingen.de>; Marcel.Werner@campus.lmu.de; Madhobi Sen <senmadhobi@gmail.com>; Taras Velychko <taras.velychko@gmail.com>; Jannasch, Katharina <katharina.jannasch@zvw.uni-goettingen.de>; Dullin, Christian <christian.dullin@med.uni-goettingen.de>; Napp, Joanna <napp@em.mpg.de>; pantel@uke.de; Wikman <h.wikman@uke.de>; Wiese, Maria <maria.wiese@med.uni-goettingen.de>; Kramm, Christof <christof.kramm@med.uni-goettingen.de>; Alves, Frauke <falves@gwdg.de>
Subject: Mieczkowska et al. - CDDIS-21-0618RR Initial Quality Check

Dear co-authors,

I just received an Email from the *Cell Death & Disease* editorial office asking me kindly to provide from all authors a statement that they agree with the accepted version of the manuscript. Therefore, I would be very happy if you could send me a short email with your confirmation so that we can proceed with the further step.
Thank you all very much.

Best wishes,

Florian

--
Dr. Florian Wegwitz
Group Leader
Laboratory for Molecular Gynecology
University Medical Center Göttingen
Department of Gynecology and Obstetrics
Georg-August-University
Robert-Koch-Straße 40
37075 Göttingen
Email: fwegwit@gwdg.de, florian.wegwitz@med.uni-goettingen.de
Tel.: +49-(0)551-39 69811

Von: Marcel Werner <Marcel.Werner@campus.lmu.de>
Gesendet: Donnerstag, 4. November 2021 14:39
An: Wegwitz, Florian
Betreff: Mieczkowska et al. - CDDIS-21-0618RR Initial Quality Check

Dear Florian,

Hereby I declare that I agree with the accepted version of the manuscript of Mieczkowska et al. for Cell Death and Disease.

Best,
Marcel Werner

Von: Madhobi Sen <senmadhobi@gmail.com>
Gesendet: Freitag, 5. November 2021 10:02
An: Wegwitz, Florian
Betreff: Re: Mieczkowska et al. - CDDIS-21-0618RR Initial Quality Check

Hi Florian,

I agree with the accepted form if the manuscript. Thank you again for including me!

Best wishes,
Madhobi

On Thu, Nov 4, 2021, 2:15 PM Wegwitz, Florian <fwegwit@gwdg.de> wrote:

> Dear co-authors,
>
> I just received an Email from the *Cell Death & Disease* editorial office asking me kindly to provide from all authors a statement that they agree with the accepted version of the manuscript. Therefore, I would be very happy if you could send me a short email with your confirmation so that we can proceed with the further step.
> Thank you all very much.
>
> Best wishes,
>
> Florian
>
> --
> Dr. Florian Wegwitz
> Group Leader
> Laboratory for Molecular Gynecology
> University Medical Center Göttingen
> Department of Gynecology and Obstetrics
> Georg-August-University
> Robert-Koch-Straße 40
> 37075 Göttingen
> Email: fwegwit@gwdg.de, florian.wegwitz@med.uni-goettingen.de
> Tel.: +49-(0)551-39 69811

Von: Taras Velychko <taras.velychko@gmail.com>
Gesendet: Donnerstag, 4. November 2021 14:24
An: Wegwitz, Florian
Betreff: Re: Mieczkowska et al. - CDDIS-21-0618RR Initial Quality Check

Dear Florian,

I agree with the accepted version of the manuscript.

Best regards,
Taras Velychko

PS: Congratulations on the paper! I am happy I could contribute to this manuscript. Thank you for that!
And warmest congratulations to Iga!

____

Taras Velychko, PhD Student
Max Planck Institute for Biophysical Chemistry
Department of Molecular Biology
Prof. Dr. Patrick Cramer
Am Faßberg 11
37077 Göttingen
Germany

E-mail: taras.velychko@mpibpc.mpg.de

On Thu, 4 Nov 2021 at 14:15, Wegwitz, Florian <fwegwit@gwdg.de> wrote:

> Dear co-authors,
>
> I just received an Email from the *Cell Death & Disease* editorial office asking me kindly to provide from all authors a statement that they agree with the accepted version of the manuscript. Therefore, I would be very happy if you could send me a short email with your confirmation so that we can proceed with the further step.
> Thank you all very much.
>
> Best wishes,
>
> Florian
>
> --
Dr. Florian Wegwitz
Group Leader
Laboratory for Molecular Gynecology
University Medical Center Göttingen
Department of Gynecology and Obstetrics
Georg-August-University
Robert-Koch-Straße 40
37075 Göttingen
Email: fwegwit@gwdg.de, florian.wegwitz@med.uni-goettingen.de
Tel.: +49-(0)551-39 69811

Von: Jannasch, Katharina
Gesendet: Freitag, 5. November 2021 08:11
An: Wegwitz, Florian
Betreff: AW: Mieczkowska et al. - CDDIS-21-0618RR Initial Quality Check

Dear Florian,

I agree with the accepted version of the manuscript.

Best regards,
Katharina

Von: Wegwitz, Florian <fwegwit@gwdg.de>
Gesendet: Donnerstag, 4. November 2021 14:15
An: Iga Mieczkowska <iga.mieczkowska92@gmail.com>; Pantelaiou Prokaki, Garyfallia <garyfallia.prokaki@em.mpg.de>; Prokakis, Evangelos <eprokak@gwdg.de>; Schmidt, Geske Elisabeth <geske.schmidt@med.uni-goettingen.de>; Müller-Kirschbaum, Lukas Christoph <lukas.mueller-kirschbaum@med.uni-goettingen.de>; Marcel.Werner@campus.lmu.de; Madhobi Sen <senmadhobi@gmail.com>; Taras Velychko <taras.velychko@gmail.com>; Jannasch, Katharina <katharina.jannasch@zvw.uni-goettingen.de>; Dullin, Christian <christian.dullin@med.uni-goettingen.de>; Napp, Joanna <napp@em.mpg.de>; pantel@uke.de; Wikman <h.wikman@uke.de>; Wiese, Maria <maria.wiese@med.uni-goettingen.de>; Kramm, Christof <christof.kramm@med.uni-goettingen.de>; Alves, Frauke <falves@gwdg.de>
Betreff: Mieczkowska et al. - CDDIS-21-0618RR Initial Quality Check

Dear co-authors,

I just received an Email from the *Cell Death & Disease* editorial office asking me kindly to provide from all authors a statement that they agree with the accepted version of the manuscript. Therefore, I would be very happy if you could send me a short email with your confirmation so that we can proceed with the further step.
Thank you all very much.

Best wishes,

Florian

--
Dr. Florian Wegwitz
Group Leader
Laboratory for Molecular Gynecology
University Medical Center Göttingen
Department of Gynecology and Obstetrics
Georg-August-University
Robert-Koch-Straße 40
37075 Göttingen
Email: fwegwit@gwdg.de, florian.wegwitz@med.uni-goettingen.de
Tel.: +49-(0)551-39 69811

Von: Dullin, Christian <christian.dullin@med.uni-goettingen.de>
Gesendet: Freitag, 5. November 2021 21:09
An: Wegwitz, Florian
Betreff: AW: PRC2 Manuscript

Dear Florian, I agree with the accepted version of the manuscript. Cheers Christian

PD Dr. sc. hum. Christian Dullin
Institute for Diagnostic and Interventional Radiology University Medical Center Goettingen Robert-Koch-Str. 40
37075 Goettingen
Germany

Von: Wegwitz, Florian [fwegwit@gwdg.de]
Gesendet: Freitag, 5. November 2021 13:49
An: Dullin, Christian
Betreff: PRC2 Manuscript

Hi Christian,

ich hoffe, es geht Dir gut!
Wie Du bestimmt gesehen hast, ist unseren Manuskript angenommen worden!
Um den Publikationsvorgang fortzufahren, brauche ich von allen Autoren eine kurze Bestätigung, dass sie mit der akzeptierten Version des Manuskripts einverstanden sind (am besten als Antwort zu meiner Email von gestern). Magst Du mir bitte das beim Gelegenheit schicken (einen Satz auf English reicht schon).
Danke Dir im Voraus.

Liebe Grüße und ein schönes WE!

Florian

--
Dr. Florian Wegwitz
Group Leader
Laboratory for Molecular Gynecology
University Medical Center Göttingen
Department of Gynecology and Obstetrics
Georg-August-University
Robert-Koch-Straße 40
37075 Göttingen
Email: fwegwit@gwdg.de<mailto:fwegwit@gwdg.de>, florian.wegwitz@med.uni-goettingen.de<mailto:florian.wegwitz@med.uni-goettingen.de>
Tel.: +49-(0)551-39 69811

Von: Napp, Joanna
Gesendet: Donnerstag, 4. November 2021 16:19
An: Wegwitz, Florian; Iga Mieczkowska; Pantelaiou Prokaki, Garyfallia; Prokakis, Evangelos; Schmidt, Geske Elisabeth; Müller-Kirschbaum, Lukas Christoph; Marcel.Werner@campus.lmu.de; Madhobi Sen; Taras Velychko; Jannasch, Katharina; Dullin, Christian; pantel@uke.de; Wikman; Wiese, Maria; Kramm, Christof; Alves, Frauke
Betreff: AW: Mieczkowska et al. - CDDIS-21-0618RR Initial Quality Check

Dear Florian,

I agree wiwth the submitted version of the manuscript, thank you!

Mit bestem Gruß,
Best wishes,

Joanna Napp

Dr. Joanna Napp

University Medical Center Göttingen (UMG)
Junior group "Image-guided Nanotherapy and Diagnostic (IND)"

Institute of Diagn. and Interv. Radiology
Robert Koch Str. 40
37075 Göttingen

MPI of exp. Medicine
Hermann Rein Str. 3
37075 Göttingen

Tel: +49 (0)551 3899272

Von: Wegwitz, Florian
Gesendet: Donnerstag, 4. November 2021 15:28:15
An: Iga Mieczkowska; Pantelaiou Prokaki, Garyfallia; Prokakis, Evangelos; Schmidt, Geske Elisabeth; Müller-Kirschbaum, Lukas Christoph; Marcel.Werner@campus.lmu.de; Madhobi Sen; Taras Velychko; Jannasch, Katharina; Dullin, Christian; Napp, Joanna; pantel@uke.de; Wikman; Wiese, Maria; Kramm, Christof; Alves, Frauke
Betreff: AW: Mieczkowska et al. - CDDIS-21-0618RR Initial Quality Check

Dear all,

Sorry, the files were missing in the previous email.
Attached the accepted manuscript files (these are the same as for the resubmission).
I wish a good afternoon!

Best regards,

Florian Wegwitz

--
Dr. Florian Wegwitz
Group Leader
Laboratory for Molecular Gynecology
University Medical Center Göttingen
Department of Gynecology and Obstetrics
Georg-August-University
Robert-Koch-Straße 40
37075 Göttingen
Email: fwegwit@gwdg.de, florian.wegwitz@med.uni-goettingen.de
Tel.: +49-(0)551-39 69811

Von: Wegwitz, Florian
Gesendet: Donnerstag, 4. November 2021 14:15
An: 'Iga Mieczkowska' <iga.mieczkowska92@gmail.com>; Pantelaiou Prokaki, Garyfallia <garyfallia.prokaki@em.mpg.de>; Prokakis, Evangelos <eprokak@gwdg.de>; Schmidt, Geske Elisabeth <geske.schmidt@med.uni-goettingen.de>; Müller-Kirschbaum, Lukas Christoph <lukas.mueller-kirschbaum@med.uni-goettingen.de>; 'Marcel.Werner@campus.lmu.de' <Marcel.Werner@campus.lmu.de>; 'Madhobi Sen' <senmadhobi@gmail.com>; 'Taras Velychko' <taras.velychko@gmail.com>; Jannasch, Katharina <katharina.jannasch@zvw.uni-goettingen.de>; Dullin, Christian <christian.dullin@med.uni-goettingen.de>; Napp, Joanna <napp@em.mpg.de>; 'pantel@uke.de' <pantel@uke.de>; 'Wikman' <h.wikman@uke.de>; Wiese, Maria <maria.wiese@med.uni-goettingen.de>; Kramm, Christof <christof.kramm@med.uni-goettingen.de>; Alves, Frauke <falves@gwdg.de>
Betreff: Mieczkowska et al. - CDDIS-21-0618RR Initial Quality Check

Dear co-authors,

I just received an Email from the *Cell Death & Disease* editorial office asking me kindly to provide from all authors a statement that they agree with the accepted version of the manuscript. Therefore, I would be very happy if you could send me a short email with your confirmation so that we can proceed with the further step.
Thank you all very much.

Best wishes,

Florian

--
Dr. Florian Wegwitz
Group Leader
Laboratory for Molecular Gynecology
University Medical Center Göttingen
Department of Gynecology and Obstetrics
Georg-August-University
Robert-Koch-Straße 40
37075 Göttingen
Email: fwegwit@gwdg.de, florian.wegwitz@med.uni-goettingen.de
Tel.: +49-(0)551-39 69811

Von: Pantel, Klaus <pantel@uke.de>
Gesendet: Freitag, 5. November 2021 12:43
An: Wegwitz, Florian; Iga Mieczkowska; Pantelaiou Prokaki, Garyfallia; Prokakis, Evangelos; Schmidt, Geske Elisabeth; Müller-Kirschbaum, Lukas Christoph; Marcel.Werner@campus.lmu.de; Madhobi Sen; Taras Velychko; Jannasch, Katharina; Dullin, Christian; Napp, Joanna; Wikman-Kocher, Harriet; Wiese, Maria; Kramm, Christof; Alves, Frauke
Betreff: AW: Mieczkowska et al. - CDDIS-21-0618RR Initial Quality Check

Dear Florian, I agree with the accepted version of the manuscript. Best wishes , Klaus

---

Von: Wegwitz, Florian <fwegwit@gwdg.de>
Gesendet: Donnerstag, 4. November 2021 14:15
An: Iga Mieczkowska; Pantelaiou Prokaki, Garyfallia; Prokakis, Evangelos; Schmidt, Geske Elisabeth; Müller-Kirschbaum, Lukas Christoph; Marcel.Werner@campus.lmu.de; Madhobi Sen; Taras Velychko; Jannasch, Katharina; Dullin, Christian; Napp, Joanna; Pantel, Klaus; Wikman-Kocher, Harriet; Wiese, Maria; Kramm, Christof; Alves, Frauke
Betreff: Mieczkowska et al. - CDDIS-21-0618RR Initial Quality Check

Dear co-authors,

I just received an Email from the *Cell Death & Disease* editorial office asking me kindly to provide from all authors a statement that they agree with the accepted version of the manuscript. Therefore, I would be very happy if you could send me a short email with your confirmation so that we can proceed with the further step.
Thank you all very much.

Best wishes,

Florian

--
Dr. Florian Wegwitz
Group Leader
Laboratory for Molecular Gynecology
University Medical Center Göttingen
Department of Gynecology and Obstetrics
Georg-August-University
Robert-Koch-Straße 40
37075 Göttingen
Email: fwegwit@gwdg.de, florian.wegwitz@med.uni-goettingen.de
Tel.: +49-(0)551-39 69811

Universitätsklinikum Hamburg-Eppendorf; Körperschaft des öffentlichen Rechts; Gerichtsstand: Hamburg | www.uke.de
Vorstandsmitglieder: Prof. Dr. Burkhard Göke (Vorsitzender), Joachim Prölß, Prof. Dr. Blanche Schwappach-Pignataro, Marya Verdel

SAVE PAPER - THINK BEFORE PRINTING

Von: Harriet Wikman <h.wikman@uke.de>
Gesendet: Donnerstag, 4. November 2021 16:38
An: Wegwitz, Florian; 'Iga Mieczkowska'; Pantelaiou Prokaki, Garyfallia; Prokakis, Evangelos; Schmidt, Geske Elisabeth; Müller-Kirschbaum, Lukas Christoph; Marcel.Werner@campus.lmu.de; 'Madhobi Sen'; 'Taras Velychko'; Jannasch, Katharina; Dullin, Christian; Napp, Joanna; pantel@uke.de; Wiese, Maria; Kramm, Christof; Alves, Frauke
Betreff: AW: Mieczkowska et al. - CDDIS-21-0618RR Initial Quality Check

Dear Florian,

I am very grateful for this great collaboration and I agree with the accepted version of the manuscript.

Best regards
Harriet

Prof. Dr. Harriet Wikman
scientist
University Medical Center Hamburg-Eppendorf
Institute of Tumor Biology
Martinistrasse 52
Building N27 / Room 04.006
20246 Hamburg
Telephone: +49 (0) 40 7410-51913
Fax: +49 (0) 40 7410-55374
h.wikman@uke.de
www.uke.de/tumorbiologie/wikman

Von: Wegwitz, Florian <fwegwit@gwdg.de>
Gesendet: 4 November, 2021 14:15 PM
An: Iga Mieczkowska <iga.mieczkowska92@gmail.com>; Pantelaiou Prokaki, Garyfallia <garyfallia.prokaki@em.mpg.de>; Prokakis, Evangelos <eprokak@gwdg.de>; Schmidt, Geske Elisabeth <geske.schmidt@med.uni-goettingen.de>; Müller-Kirschbaum, Lukas Christoph <lukas.mueller-kirschbaum@med.uni-goettingen.de>; Marcel.Werner@campus.lmu.de; Madhobi Sen <senmadhobi@gmail.com>; Taras Velychko <taras.velychko@gmail.com>; Jannasch, Katharina <katharina.jannasch@zvw.uni-goettingen.de>; Dullin, Christian <christian.dullin@med.uni-goettingen.de>; Napp, Joanna <napp@em.mpg.de>; pantel@uke.de; Wikman <h.wikman@uke.de>; Wiese, Maria <maria.wiese@med.uni-goettingen.de>; Kramm, Christof <christof.kramm@med.uni-goettingen.de>; Alves, Frauke <falves@gwdg.de>
Betreff: Mieczkowska et al. - CDDIS-21-0618RR Initial Quality Check

Dear co-authors,

I just received an Email from the *Cell Death & Disease* editorial office asking me kindly to provide from all authors a statement that they agree with the accepted version of the manuscript. Therefore, I would be very happy if you could send me a short email with your confirmation so that we can proceed with the further step.
Thank you all very much.

Best wishes,

Florian

--
Dr. Florian Wegwitz
Group Leader
Laboratory for Molecular Gynecology
University Medical Center Göttingen
Department of Gynecology and Obstetrics
Georg-August-University
Robert-Koch-Straße 40
37075 Göttingen
Email: fwegwit@gwdg.de, florian.wegwitz@med.uni-goettingen.de
Tel.: +49-(0)551-39 69811

Universitätsklinikum Hamburg-Eppendorf; Körperschaft des öffentlichen Rechts; Gerichtsstand: Hamburg | www.uke.de
Vorstandsmitglieder: Prof. Dr. Burkhard Göke (Vorsitzender), Joachim Prölß, Prof. Dr. Blanche Schwappach-Pignataro, Marya Verdel

---

SAVE PAPER - THINK BEFORE PRINTING

Von: Wiese, Maria <maria.wiese@med.uni-goettingen.de>
Gesendet: Samstag, 6. November 2021 16:28
An: Wegwitz, Florian; Iga Mieczkowska; Pantelaiou Prokaki, Garyfallia; Prokakis, Evangelos; Schmidt, Geske Elisabeth; Müller-Kirschbaum, Lukas Christoph; Marcel.Werner@campus.lmu.de; Madhobi Sen; Taras Velychko; Jannasch, Katharina; Dullin, Christian; Napp, Joanna; pantel@uke.de; Wikman; Kramm, Christof; Alves, Frauke
Betreff: AW: Mieczkowska et al. - CDDIS-21-0618RR Initial Quality Check

Dear Florian,
I agree with the accepted version of the manuscript.
Thank you very much and best wishes,
Maria

Dr. Maria Wiese
Experimental Pediatric Neurooncology
Division of Pediatric Hematology and Oncology
Department of Pediatrics and Adolescent Medicine
University Medical Center Goettingen
Robert Koch Straße 40
37075 Goettingen, Germany

Phone: +49-551-39-62280 (Office)/ -22575/10568 (Lab)

Von: Wegwitz, Florian <fwegwit@gwdg.de>
Gesendet: Donnerstag, 4. November 2021 14:15
An: Iga Mieczkowska <iga.mieczkowska92@gmail.com>; Pantelaiou Prokaki, Garyfallia <garyfallia.prokaki@em.mpg.de>; Prokakis, Evangelos <eprokak@gwdg.de>; Schmidt, Geske Elisabeth <geske.schmidt@med.uni-goettingen.de>; Müller-Kirschbaum, Lukas Christoph <lukas.mueller-kirschbaum@med.uni-goettingen.de>; Marcel.Werner@campus.lmu.de; Madhobi Sen <senmadhobi@gmail.com>; Taras Velychko <taras.velychko@gmail.com>; Jannasch, Katharina <katharina.jannasch@zvw.uni-goettingen.de>; Dullin, Christian <christian.dullin@med.uni-goettingen.de>; Napp, Joanna <napp@em.mpg.de>; pantel@uke.de; Wikman <h.wikman@uke.de>; Wiese, Maria <maria.wiese@med.uni-goettingen.de>; Kramm, Christof <christof.kramm@med.uni-goettingen.de>; Alves, Frauke <falves@gwdg.de>
Betreff: Mieczkowska et al. - CDDIS-21-0618RR Initial Quality Check

Dear co-authors,

I just received an Email from the *Cell Death & Disease* editorial office asking me kindly to provide from all authors a statement that they agree with the accepted version of the manuscript. Therefore, I would be very happy if you could send me a short email with your confirmation so that we can proceed with the further step.
Thank you all very much.

Best wishes,

Florian

--
Dr. Florian Wegwitz
Group Leader
Laboratory for Molecular Gynecology
University Medical Center Göttingen
Department of Gynecology and Obstetrics
Georg-August-University
Robert-Koch-Straße 40
37075 Göttingen
Email: fwegwit@gwdg.de, florian.wegwitz@med.uni-goettingen.de
Tel.: +49-(0)551-39 69811

Von: Kramm, Christof
Gesendet: Freitag, 5. November 2021 17:05
An: Wegwitz, Florian; Wegwitz, Florian
Betreff: I agree to the current manuscript version

Thanks and best wishes
Christof

Christof Kramm via iPhone

Von: Alves, Frauke
Gesendet: Donnerstag, 4. November 2021 16:39
An: Wegwitz, Florian
Betreff: Mieczkowska et al. - CDDIS-21-0618RR Initial Quality Check

Dear Florian,

I agree with the submitted and accepted version of the manuscript!

Decreased PRC2 activity supports the survival of basal-like breast cancer cells to cytotoxic treatments.

by Iga K. Mieczkowska[1*], Garyfallia Pantelaiou-Prokaki[2, 3*], Evangelos Prokakis[2], Geske E. Schmidt[4], Lukas C. Müller-Kirschbaum[1], Marcel Werner[1], Madhobi Sen[1], Taras Velychko[1], Katharina Jannasch[5], Christian Dullin[3, 5, 6], Joanna Napp[3, 6], Klaus Pantel[7], Harriet Wikman[7], Maria Wiese[8], Christof M. Kramm[8], Frauke Alves[3, 5, 6], Florian Wegwitz

with best regards

Frauke Alves

Prof. Dr. med. Frauke Alves

Translational Molecular Imaging

Institute of Diagnostic and Interventional Radiology
Clinic of Hematology and Medical Oncology
University Medical Center Goettingen (UMG)
Robert Koch Str. 40
37075 Göttingen
Tel: +49 (0)551 39 66991

&
MPI for exp. Medizin
Hermann Rein Str. 3
37075 Göttingen
Tel: +49 (0)551 3899 655

Von: Wegwitz, Florian
Gesendet: Donnerstag, 4. November 2021 14:15
An: Iga Mieczkowska; Pantelaiou Prokaki, Garyfallia; Prokakis, Evangelos; Schmidt, Geske Elisabeth; Müller-Kirschbaum, Lukas Christoph; Marcel.Werner@campus.lmu.de; Madhobi Sen; Taras Velychko; Jannasch, Katharina; Dullin, Christian; Napp, Joanna; pantel@uke.de; Wikman; Wiese, Maria; Kramm, Christof; Alves, Frauke
Betreff: Mieczkowska et al. - CDDIS-21-0618RR Initial Quality Check

Dear co-authors,

I just received an Email from the *Cell Death & Disease* editorial office asking me kindly to provide from all authors a statement that they agree with the accepted version of the manuscript. Therefore, I would be very happy if you could send me a short email with your confirmation so that we can proceed with the further step.
Thank you all very much.

Best wishes,

Florian

--
Dr. Florian Wegwitz
Group Leader
Laboratory for Molecular Gynecology
University Medical Center Göttingen
Department of Gynecology and Obstetrics
Georg-August-University
Robert-Koch-Straße 40
37075 Göttingen
Email: fwegwit@gwdg.de, florian.wegwitz@med.uni-goettingen.de
Tel.: +49-(0)551-39 69811
